# Electromechanical Response of Saddle Points in Twisted hBN Moiré Superlattices

**DOI:** 10.1021/acsnano.4c12315

**Published:** 2025-04-23

**Authors:** Stefano Chiodini, Giacomo Venturi, James Kerfoot, Jincan Zhang, Evgeny M. Alexeev, Takashi Taniguchi, Kenji Watanabe, Andrea C. Ferrari, Antonio Ambrosio

**Affiliations:** † Center for Nano Science and Technology, Fondazione Istituto Italiano di Tecnologia, Via Rubattino 81, 20134 Milan, Italy; ‡ Cambridge Graphene Centre, University of Cambridge, 9, JJ Thomson Avenue, CB3 0FA Cambridge, United Kingdom; § Center for Materials Nanoarchitectonics, National Institute for Materials Science, 1-1 Namiki, Tsukuba 305-0044, Japan; ∥ Research Center for Functional Materials, National Institute for Materials Science, 1-1 Namiki, Tsukuba 305-0044, Japan

**Keywords:** moiré superlattices, hexagonal boron nitride, piezo force microscopy, electromechanics, saddle points

## Abstract

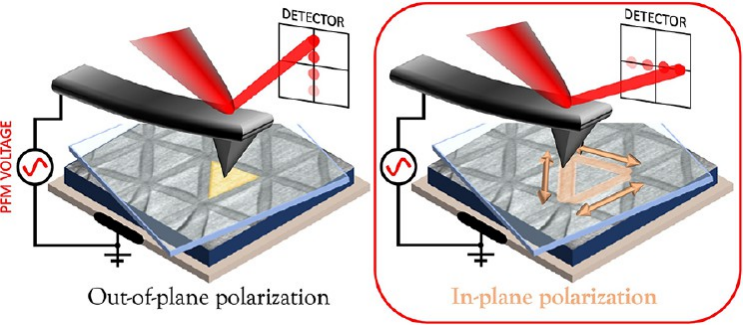

In twisted layered materials (t-LMs), an interlayer rotation can break inversion symmetry and create an interfacial array of staggered out-of-plane polarization due to AB/BA stacking registries. This symmetry breaking can also trigger the formation of edge *in-plane* polarizations localized along the perimeter of AB/BA regions (i.e., saddle point domains). However, a comprehensive experimental investigation of these features is still lacking. Here, we use piezo force microscopy to probe the electromechanical behavior of twisted hexagonal boron nitride (t-hBN). For parallel stacking alignment of t-hBN, we reveal very narrow (width ∼ 10 nm) saddle point in-plane polarizations, which we also measure in the antiparallel configuration. These localized polarizations can still be found on a multiply stacked t-hBN structure, determining the formation of a double moiré. Our findings imply that polarizations in t-hBN do not only point in the out-of-plane direction but also show an in-plane component, giving rise to a much more complex 3D polarization field.

The detection and manipulation of electric,^1^ magnetic^2^ and valley polarizations^3^ are key for device performance optimizations.^4^ As Moore’s law approaches its physical limits,^4^ the need for miniaturized nanoelectronics,^5^ involving high-density integrated circuits and low power consumption^6^ has triggered research into layered materials (LMs),^7,8^ in order to reduce polarization domains from the 100 nm^2^ scale down to the atomic scale.^5^ Room temperature out-of-plane ferroelectricity offers a wide range of technological applications, such as ultrathin nonvolatile memories^9^ and high-permittivity dielectrics.^9,10^ However, only few suitable ferroelectric LMs have been identified so far, like CuInP_2_S_6_,^11^ In_2_Se_3_,^12,13^ MoTe_2_^14^ and WTe_2_^15^ in their 1T phase.

In other widely studied LMs, such as hexagonal boron nitride (hBN) and 2H-type transition metal dichalcogenides (TMDs), vertical polarizations cancel out,^16^ due to the centrosymmetric lattice structure, which makes these crystals unpolarized. A possible way to engineer polarization in these LMs is to break the inversion symmetry by introducing a twist angle, θ_TW_, between top and bottom layers,^16−18^ determining a periodic modulation of the interlayer atomic registry, i.e., a moiré superlattice. In twisted hBN (t-hBN) structures the interfacial vertical alignment of the N and B atoms distorts the bonding *2p*_*z*_ N electronic orbital,^17^ locally creating an electric dipole moment that leads to a moiré superlattice characterized by adjacent domains with out-of-plane (OOP) polarizations pointing in opposite directions.^16−19^ Refs (18), (20), and (21) predicted that in-plane (IP) polarizations can also appear at the moiré domains’ *edges* of t-hBN (with clockwise or anticlockwise orientation), resulting into three dimensional (3D) vectorial patterns with rich topological structures. Topology plays a key role in LMs, ranging from band theory to skyrmions in magnetic samples.^20^ Topological domains in ferroelectrics^22−24^ received much attention, owing to their novel functionalities, such as negative capacitance^25^ and high-density information processing.^26^ However, experimental proofs of the IP polarizations in t-LMs are limited to irregular t-hBN moiré patterns,^27^ or twisted double bilayer graphene samples.^28^

Here, we use piezo force microscopy (PFM) to reveal edge IP polarizations in t-hBN moiré superlattices for parallel and antiparallel stacking. We find very sharp (width ∼ 10 nm) polarizations localized at the edges between different domains of the moiré pattern, called saddle points (SPs), not seen by other scanning probe microscopy (SPM) techniques, such as electrostatic force microscopy (EFM),^18,29^ amplitude-modulation kelvin probe force microscopy (AM-KPFM),^18^ and tapping mode phase imaging.^30^ We prove the universality of these SP features by systematically probing them for superlattices corresponding to different in the range 0.04–0.18°. We also explore samples consisting of three hBN stacks (i.e., two twisted interfaces).^31^ The superposition of SP polarizations arising at the two interfaces is still measurable by PFM, showing a double moiré. The possibility of interfacing multiple layer polarizations could pave the way for unconventional properties, such as modulations of moiré ferroelectric behaviors.

## Results and Discussion

### Parallel Stacking Alignment in t-hBN

**Figure 1:**
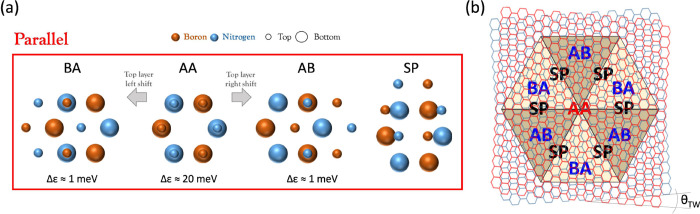
t-hBN parallel stacking configurations. (a) Atomic registries corresponding to the 4 domains (AA, AB, BA, SP) typical of parallel stacking in a t-hBN interface (for the SP region we illustrate the average atomic registry). For AA, AB, BA configurations, the corresponding stacking energy per atom, Δε, relative to the naturally occurring AA’ registry,^32^ is reported. B and N atoms of top (smaller circles) and bottom (larger circles) layers are sketched in maroon and blue, respectively. (b) Representation of two hBN atomic layers, red and blue, (rigidly) stacked and twisted by a small θ_TW_ < 1° . The superimposed internal drawing represents the typical 6 triangular shapes obtained after atomic relaxation, defining the moiré superlattice. The position of each of the 4 domains (AA, AB, BA, SP) is shown.

When two hBN layers are stacked together and twisted, the misalignment of the rotated atoms results in a periodic array of local stacking domains, i.e., a moiré superlattice.^18^ To rationalize the geometry of t-hBN stacking domains and their 3D polarization network (IP and OOP), the hBN unit cell has to be considered. For t-hBN, two stacking alignments are possible, i.e., parallel and antiparallel.^18,21,33^ For parallel stacking, 4 different domains can be identified: AA, AB, BA, SP. Their specific atomic registry is reported in Figure 1a. The AA configuration is characterized by a full overlap between N (B) atoms of one layer and N (B) atoms of the twisted layer. In AB (BA) registry, the B (N) atoms in the top layer sit above the N (B) atoms in the bottom layer, while the N (B) atoms in the upper layer lay above the empty site at the center of the hexagonal cell of the lower layer. SP regions are between different domains, where the atomic registry changes from one domain to another.

The alternation of these 4 stacking regions forms the parallel moiré superlattice (where “parallel” refers to the stacking alignment) of t-hBN (Figure 1b), according to a geometry which is set by a -dependent balance^16^ between interlayer interactions and intralayer elasticity of the lattice, i.e., the atomic relaxation. This is the driving force shaping the moiré domains’ geometry (triangular or hexagonal), mainly at θ_TW_ < 1°, where atomic relaxation is more pronounced).^16−18,34^

According to simulations,^32^ AB and BA regions are energetically equivalent with a corresponding stacking energy (calculated with respect to the natural AA’ stacking configuration)^32^ Δε∼ 1 meV (Figure 1a) and, most importantly, energetically favorable with respect to the AA domain (Δε∼ 20 meV), since the latter has pairs of N atoms atop of each other, resulting in an increased steric repulsion.^16^ Hence, as shown in Figure 1b, for parallel alignment at θ_TW_ < 1°, AB/BA regions cover the majority of the moiré superlattice, with a triangular geometry,^18,21,33^ while unfavorable AA domains are reduced to a smaller hexagonal coverage (Figure 3f).^21,27,35,36^

In Supporting Information (SI), Section 1, we extend the description of the stacking domains to the t-hBN antiparallel alignment.

### PFM of t-hBN Parallel Moiré Superlattices

We first consider a 2 nm thick top hBN (∼5 layers) on an 8 nm bottom hBN (>10 layers) on Si + 285 nm SiO_2_. The two flakes are aligned at θ_TW_ ∼ 0°. This sample is characterized by PFM, where a conductive tip is in contact with the surface, while an oscillating electrical bias is applied via the tip itself. Through PFM, the electromechanical (EM) response can be measured.^37^ We define the EM coupling as any effect that produces an electric field across the material in response to a surface or volume deformation and vice versa (i.e., piezoelectric and inverse-piezoelectric effects,^37^ respectively). Due to the inverse piezoelectric effect, an electromechanically active sample deforms under a bias, and this distortion couples with the cantilever motion, whose deflection is measured by the cantilever detection system (i.e., the standard AFM optical lever system - such as for our microscope - or the more powerful interferometric displacement sensor).^37^ More details in SI, Section 2. The origin of this EM sample deformation can arise from two main effects, piezoelectricity (PZ) or flexoelectricity (FLX).^38^ PZ allows conversion of mechanical *strain* into electric fields (and vice versa) and it arises only in noncentrosymmetric samples, i.e., when a broken inversion symmetry is present.^39^ FLX, instead, allows a material to polarize in response to a *strain gradient* (i.e., mechanical bending), and, conversely, to bend in response to an electric field. Despite half a century of history, the latter has been less considered because of its expected weak strength at the macroscale.^38^ However, at the nanoscale, FLX can compete with PZ, or be bigger.^38^ FLX is a universal property of all materials, without any symmetry constraint.^40^

**Figure 2:**
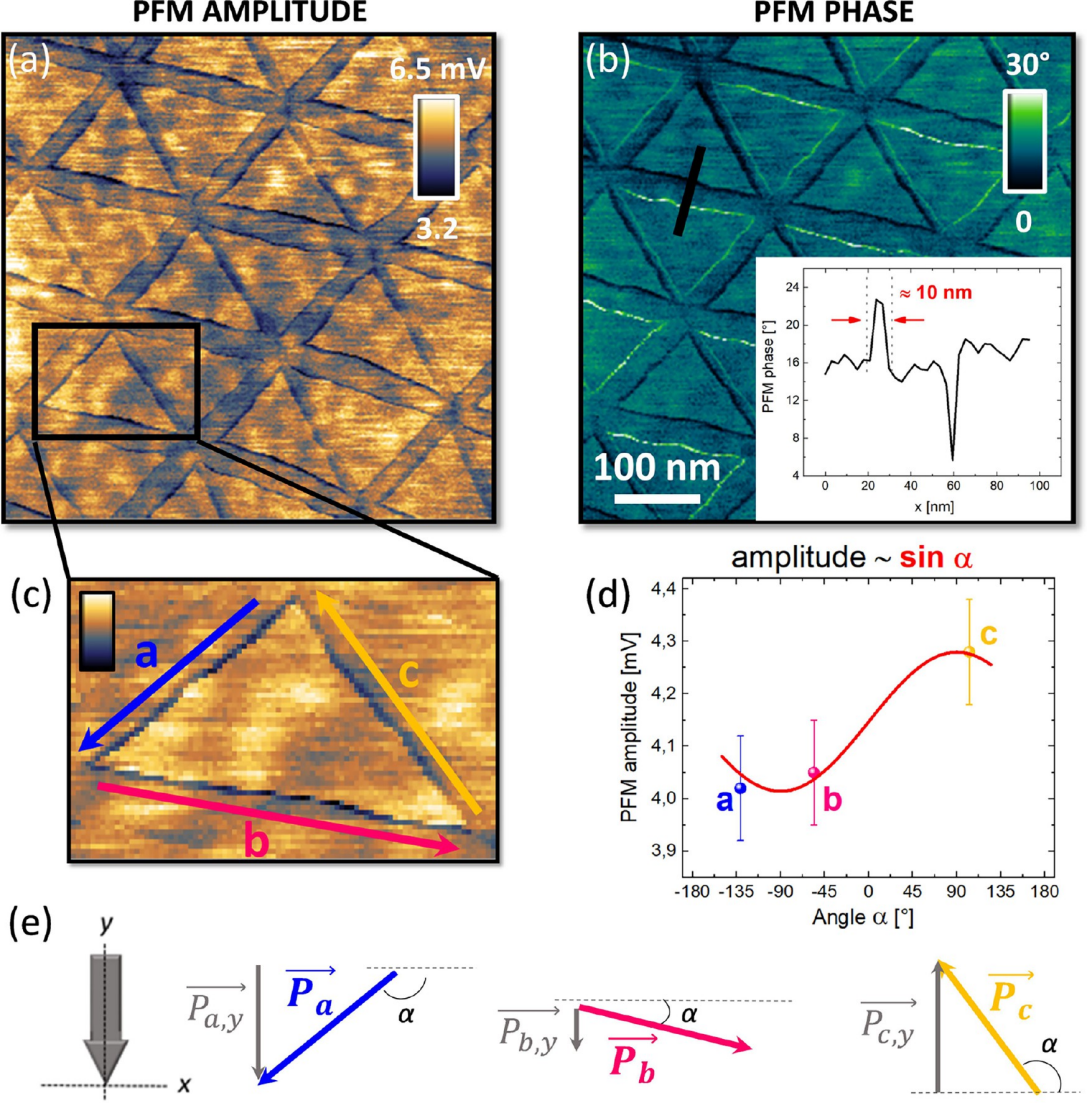
IP polarizations measurement via vertical PFM in t-hBN. (a, b) PFM amplitude and phase images of t-hBN. The inset of (b) is a PFM phase line profile highlighting a feature localized in a width ∼ 10 nm. (c) Zoom of a representative triangular domain in (a). The 3 triangle sides are labeled (a–c) following an anticlockwise orientation. (d) PFM average amplitude as a function of an angle, α, between the triangle side (a–c) and the *x*-axis (panel (e)). Data and error bars are obtained averaging over 7 triangular domains. Red line: best fit with sinusoid: *A* = *y*_0_ + *a* · sin α (*y*_0,_*a*: fitting parameters). In SI, Section 5 we report similar measurements with 9 experimental points, obtained by repeating the same PFM measurements for 3 different sample orientations. (e) Vectorial decomposition of each polarization involved in the triangular shape in (c). (Blue, pink, gold): polarization vectors . Gray: components along the *y*-axis (main cantilever axis) with *i* = *a*, *b*, *c*. α measured with respect to the positive direction of the *x*-axis. All polarizations are oriented anticlockwise.

Figure 2a,b shows two representative PFM amplitude and phase images obtained on our t-hBN sample (topography reported in SI, Section 3). The moiré domains are characterized by narrow features at the edge of the triangular AB/BA regions (width ∼ 10 nm, inset of Figure 2b), which look the same in both trace and retrace maps (see SI, Section 3). This observation points toward the reliability of the PFM signals even if artifacts could still affect this mapping,^37^ (SI, Section 3). Figure 2c is a zoom of a (representative) triangular domain from the PFM amplitude map (Figure 2a), where the 3 edges of the triangle (a, b, c) are highlighted. Since we measure the EM response of the sample via vertical PFM, one could expect these features to emerge from OOP polarizations. However, IP polarizations detection through standard vertical PFM is also possible, due to the buckling effect.^41,42^ This stems from cantilever buckling oscillations occurring when domains with IP polarization are aligned parallel to the long axis of the cantilever (see SI, Section 4). Based on this, we now prove the observed features to emerge from an IP contribution to the sample EM response. The experimental proof is provided in V-PFM (Figure 2) and L-PFM (SI, Section 5), going beyond past literature that, for t-hBN, only focused on L-PFM,^27^ a technique not always available on AFM microscopes.

If the buckling effect is relevant, the measured PFM amplitude should emerge from the vectorial coupling between the cantilever main axis and the projection of the IP polarization along this very axis. Hence, we expect an angle-dependent PFM amplitude (*A*) signal,^27^ i.e., A ∼ *P*_*i*_ · sin α = , where  is the IP polarization vector, the label *i* = *a, b, c* refers to one of the sides of a triangle (Figure 2c,e), and α is the angle between the cantilever *x*-axis and the triangle side under consideration (Figure 2e). In Figure 2e, the polarizations  are in blue, pink and gold, while their y-component in gray.

To corroborate this hypothesis, we report in Figure 2d the average (over seven triangles sides) of the PFM amplitudes measured along the triangular edges as a function of α. The three data points nicely fit a sinusoidal function, *i.e., A* = *y*_0_ + *a* · sin α, with *y*_0_ and *a* as fitting parameters, representing the global background of the PFM image and the amplitude of the oscillation, respectively. This can be considered the fingerprint of the IP nature of such polarizations localized along the SPs of the triangular moiré domains.^27^ In SI, Section 5 we increase the statistics of Figure 2d from 3 to 9 points. As shown in Figure S4j, this extended set of data is fitted by a sinusoidal, further supporting our interpretation of the IP nature of these edge polarizations.

Notably, such IP polarizations can also couple with the torsional motion of the cantilever probed in lateral PFM. As an additional confirmation of the IP nature of these SP polarizations, in SI, Section 5, we provide lateral PFM images of a t-hBN moiré pattern.

Figure 2a,b show that the internal area of the triangular domains does not offer any EM contrast between adjacent triangular regions (AB/BA domains). Refs (17) and (43) reported that nearby triangular AB/BA domains provide a PFM contrast in the internal area. In our case, the different sample thicknesses could play a role. Indeed, while we work with a 2 nm-thick top hBN flake, refs (17) and (44) used monolayer (1L) top hBN. Due to a vertical PFM sensitivity necessarily dependent on the top layer thickness, as a result of our larger flakes thicknesses, the vertical EM contrast between AB and BA polarizations is not measurable. This is confirmed by performing the same measurements on a different t-hBN sample with a 0.8 nm-thick top layer (with a bottom flake of 5.7 nm on Si + 285 nm SiO_2_). We report the corresponding V-PFM amplitude and phase channels in Section 11 of the SI, where the contrast between AB/BA domains can be appreciated in Figure S12b,c.

The EM origin of these IP polarizations (PZ and/or FLX) is still under debate.^27,45^ However, the inset of Figure 2b shows a phase profile with two opposite peaks (with respect to the common background of ∼16°), pointing toward the presence of opposite IP polarizations across the SP. Following the investigations on twisted bilayer graphene of ref (45). (Figure 4b), this could be in line with a major FLX contribution to the EM response of our sample. Nevertheless, we cannot exclude the presence of PZ effects. According to ref (45), the sample EM response is thickness dependent and, for our specific case of multilayer hBN, it can also have contributions of PZ phenomena.^45^

**Figure 3:**
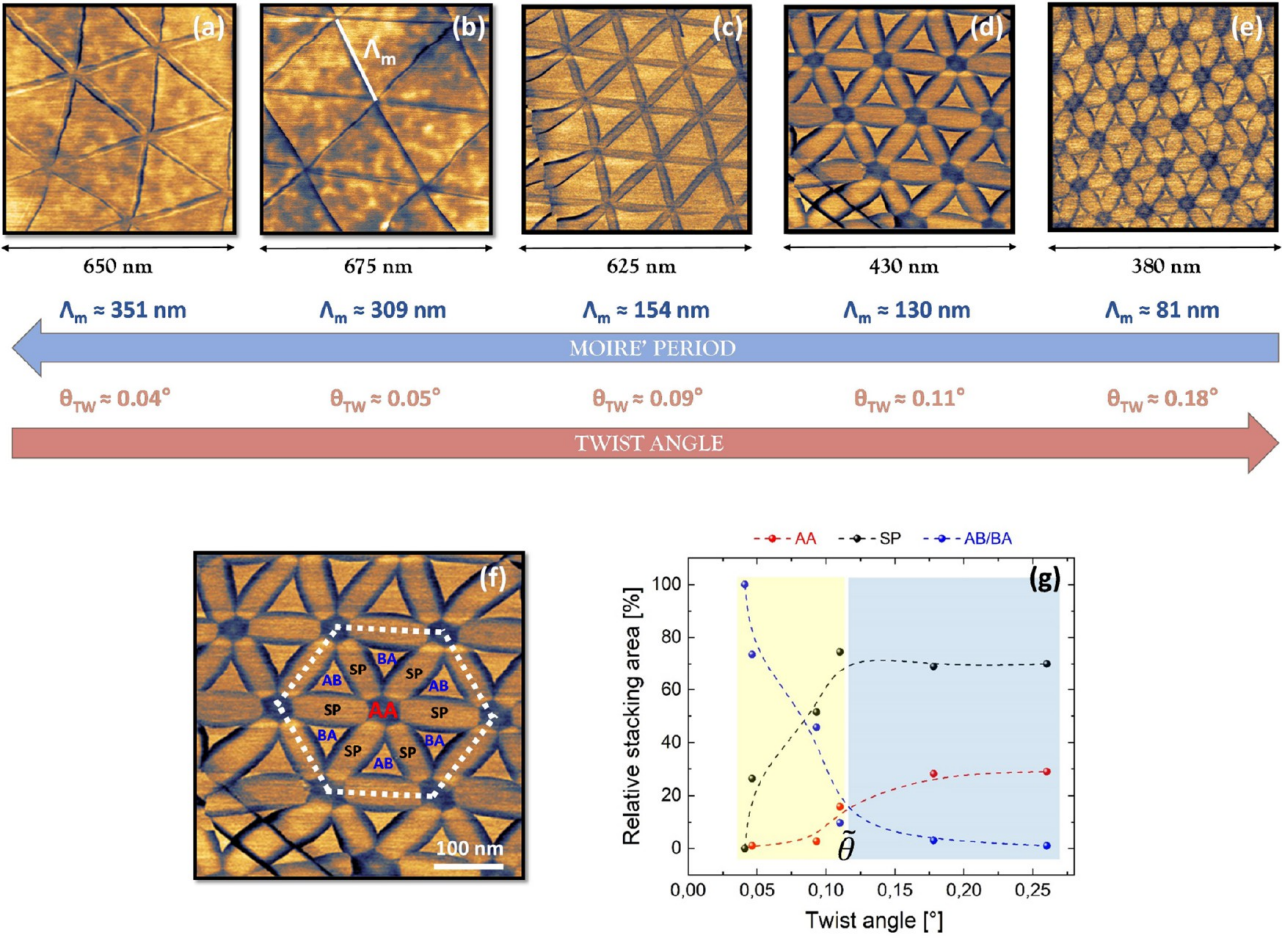
Parallel stacking domains evolution with increasing θ_TW_. (a–e) 5 moiré patterns characterized by a different moiré period (Λ_m_) corresponding to an increasing θ_TW_ between top and bottom hBN. At the bottom of each image, the scan size is reported. The big blue and red arrows show the evolution of Λ_m_ and θ_TW_, respectively. For the determination of θ_TW_, see the SI, Section 6, while Λ_m_ is experimentally determined as the average distance between AA domains.^47^ Amplitude scale bar: (a) 2.7–6.6 mV, (b) 1.3–2.3 mV, (c) 6.3 - 9.9 mV, (d) 8.5–14.5 mV, (e) 19–24 mV. (a, b) are for a t-hBN with top flake thickness ∼ 2 nm, bottom flake thickness ∼ 8 nm, θ_TW_ ∼ 0°. (c–e) are for a different t-hBN with top layer thickness ∼ 4.5 nm, bottom flake thickness ∼40 nm, θ_TW_ ∼ 0.2°. (f) PFM amplitude map (equivalent to Figure 3d), with all stacking domains identified on the surface (AB/BA, AA, SP). The white dashed line represents the moiré superhexagonal shape used in the relative stacking area quantification of (g). (g) Relative (%) stacking area evolution with increasing θ_TW_ for AA (red), SP (black) and AB/BA (blue) regions. Following the SP trend, 2 regions can be highlighted: a first one (yellow), for θ_TW_ <  ∼ 0.1°, where the SP relative area is increasing with θ_TW_, and a second (blue) where this trend saturates reaching a plateau for θ_TW_ >  0.1°. The last point on the right of the plot (θ_TW_ ∼ 0.25°) is obtained on the additional PFM image in Figure 5b, corresponding to Λ_m_ ∼ 55 nm.

We now extend the analysis of SP polarizations to moiré patterns arising from different. In order to consider them a general feature of such superlattices, they have to be present independently of θ_TW_. The fact that θ_TW_ may vary on a given sample is a consequence of the fabrication procedure, which does not allow for deterministic control of θ_TW_. Defects and fabrication residuals with unknown distribution over the sample areas can locally alter the twisted structure causing heterogeneous strain distributions and variations of θ_TW_. Hence, when dealing with a t-LM at a specific θ_TW_, we expect local deviations around the target value, which will also tune the moiré superlattice to a different periodicity (Λ_m_, see Figure 3b). According to the theory of moiré superlattices, an inverse relation exists between Λ_m_ and i.e., Λ_m_ = (*a*/2)/sin(θ_TW_/2),^28,46^ with a corresponding to the hBN lattice constant of 0.25 nm.^47^ This formula is valid under two assumptions: the hBN layers are treated as rigid (i.e., atomic relaxation is neglected, see SI, Section 6 for more information), and they are unaffected by strain. The latter constraint can be relaxed by considering the presence of strain, as in ref (46), and SI, Section 6. In our case, the θ_TW_ variation with respect to an unstrained case is calculated to be only ∼5%.

Figure 3a–e plot the PFM amplitude images obtained on different sample regions characterized by a decreasing (parallel) moiré pattern period. Λ_m_ ranges from ∼350 to ∼80 nm, corresponding to an increasing estimated θ_TW_ from ∼0.04 to ∼0.18°. For each image, sharp features are present at the evolving SP regions, revealing the universality of this localized EM response of t-hBN.

Figure 3a–e also allow us to evaluate the shape evolution of all atomic registries with θ_TW_. Their identification, from AB/BA domains (in blue) to AA (in red) and SP (in black) is presented in Figure 3f. This image proves AA domains to have a hexagonal shape, as theoretically expected,^21,36^ but, thus far, not observed experimentally, to the best of our knowledge. Going from Figure 3a–e, there is a decreasing coverage of triangular AB/BA regions, in favor of AA hexagonal domains, progressively growing in size. To quantify this evolution, we first need to define a superhexagon for each PFM image of Figure 3a–e. This acts as an effective “unit-cell” for the moiré superlattice and encloses 3 AB and 3 BA triangular domains. E.g., the superhexagon corresponding to Figure 3d is highlighted by the white dashed line in Figure 3f. Second, for each PFM amplitude image, we can obtain the areal coverage^48^ for AA, AB/BA, SP regions confined inside the corresponding superhexagon. These areas can be normalized dividing by the total coverage of the superhexagon itself, defining what we have called in Figure 3g “relative stacking area”. The θ_TW_-dependent evolution of the relative stacking area for AA, AB/BA, SP regions is illustrated in Figure 3g. Different trends are observed: while the AA relative coverage increases with θ_TW_ (red data), the AB/BA behavior (blue data) decreases. This is consistent with a θ_TW_ -dependent atomic relaxation, progressively decreasing the relative area covered by AB/BA triangular domains at larger angles, favoring AA regions.^36^

There is a point where the relative coverages of AB/BA and AA domains balance, marking the boundary between two reconstruction regimes where energetically unfavorable domains take over. This happens at θ_TW_ =  ∼ 0.10°, further confirmed by following the SP relative area evolution (black data). For θ <  we observe a linear SP trend, which then reaches a plateau for θ >  . A similar trend was reported for t-BLG,^35^ where  assumes a higher relevance as it marks the appearance of flat bands, via 4D-scanning transmission electron microscopy (4D-STEM).^35^ Hence, we believe PFM could be employed for the identification of correlated electronic states in t-LMs.

The analogous evolution of the EM response of moiré superlattices for the antiparallel alignment probed at two different θ_TW_ is presented in the SI, Section 7.

### PFM of t-hBN Antiparallel Moiré Superlattices

**Figure 4:**
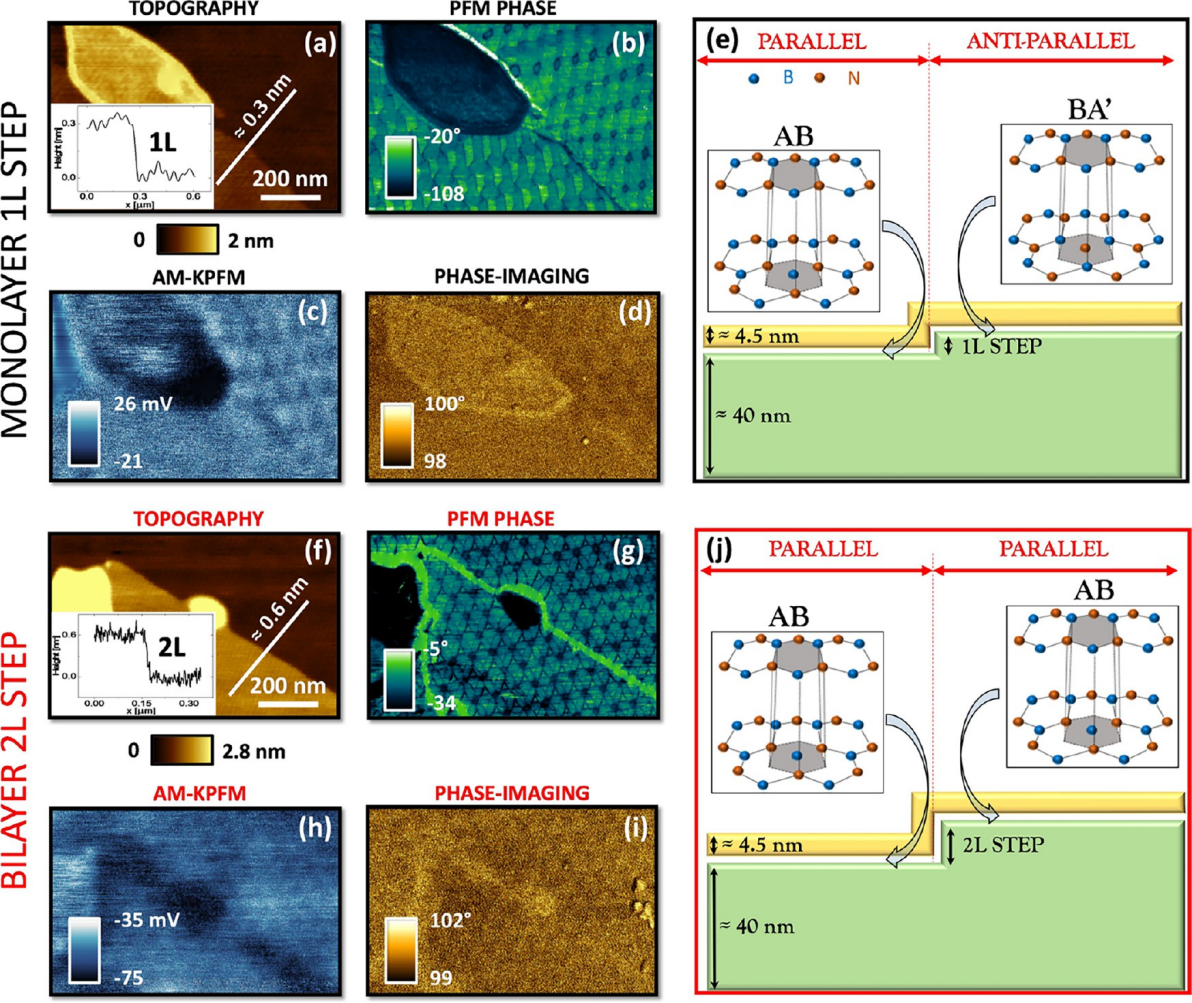
t-hBN parallel and antiparallel stacking domains measured with different SPM techniques. (a–e): Parallel to antiparallel alignment transition induced by a 1L topographical step ∼ 0.3 nm on a t-hBN sample with top flake thickness ∼ 4.5 nm, bottom flake thickness ∼ 40 nm, θ_TW_ ∼ 0.2°. (a) AFM topography of 1L step. (b–d) PFM phase, AM-KPFM, and phase-imaging maps in the same region of (a). (e) Schematic sample structure corresponding to Figure 4a–d. On the top part a possible stacking transition is shown from parallel AB to antiparallel BA’ lattice registry. (f–j): Parallel to parallel alignment transition due to a 2L-hBN topographical step ∼ 0.6 nm. (f) AFM topography of 2L-hBN step. (g–i) corresponding PFM phase, AM-KPFM, and phase-imaging channels. (j) Schematic sample structure corresponding to Figure 4f–i. The top part of panel (j) sketches a parallel stacking domain (AB) on both sides of the 2L-hBN step. The thicknesses of the flakes and steps are not to scale.

We further generalize the relevance of SP polarizations by experimentally revealing their presence also for t-hBN antiparallel stacking alignments. To access this interfacial alignment, we exploit the topography of a t-hBN sample offering a 1L step underneath the top flake, Figure 4a (see Figure 4e for a sketch of the sample structure). Since multilayer hBN (ML-hBN) such as the bottom flake, has a natural AA’ stacking,^18^ the addition of a 1L step, would produce a rotation of 180° with respect to the underlying structure, determining a parallel to antiparallel stacking transition (Figure 4e).^18^Figure 4a confirms the step to correspond to 1L of hBN, ∼0.3 nm.^18^Figure 4b plots the related PFM phase (see SI, Section 8, for the corresponding amplitude map). While triangular AB/BA domains are visible on the top-right part of these three images (parallel interfacial stacking), the bottom-left region, corresponding to the 1L-hBN addition, shows hexagonal structures typical of *anti-parallel* stacking, with features localized at the SP domains (see also Figure S8d). Figure 4c,d show the corresponding AM-KPFM and phase-imaging maps of the same region. While all three SPM techniques allow the visualization of AB/BA triangular domains, PFM is the only approach capable of visualizing antiparallel stacking. The reason for this can only stem from the different imaging mechanisms. Indeed, while PFM relies on the EM coupling between tip and sample, AM-KPFM and phase-imaging are noncontact AFM techniques probing their electrostatic interaction.

To validate this further, we focus on a different region of the same sample offering a topographical 2L-hBN step ∼ 0.6 nm (see Figure 4f for the topography and Figure 4j for the sample structure). If a 1L-hBN step is responsible for a 180° rotation, it follows that a 2L-hBN step does not induce any parallel to antiparallel stacking transition. Figure 4g–i shows the PFM phase (PFM amplitude image shown in the SI, Section 8), AM-KPFM, and phase-imaging maps of the same region, addressing a parallel stacking on both sides of the 2L-hBN step.

### Double-Moiré

**Figure 5:**
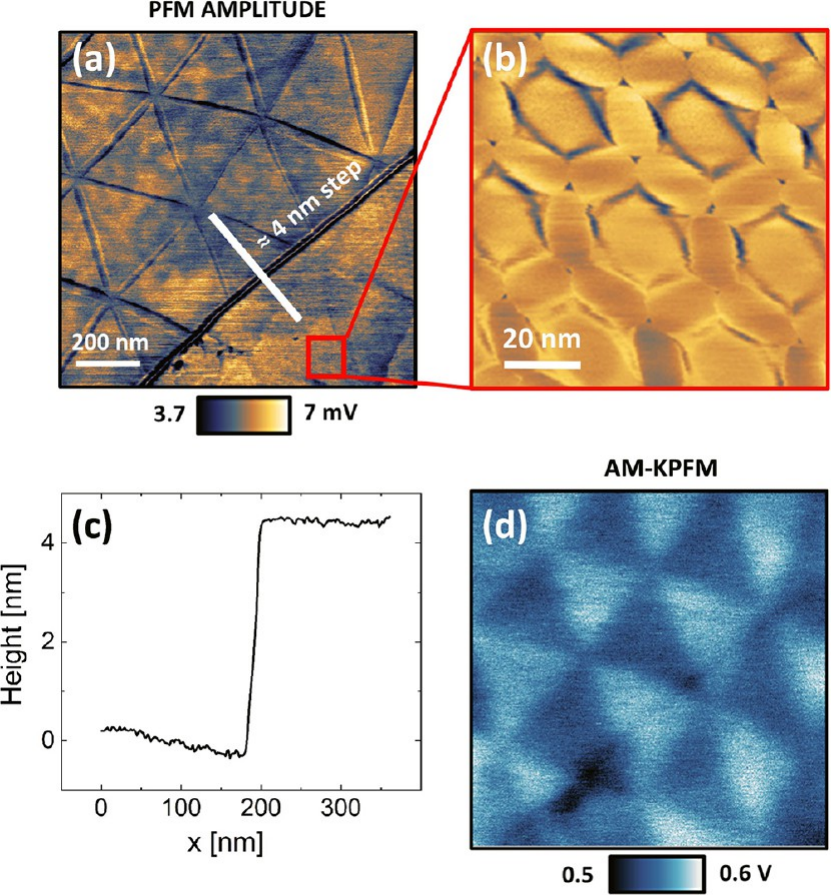
Double-moiré in multiply stacked t-hBN measured via PFM. (a) PFM amplitude map showing a double-moiré in the bottom-right corner. (b) Zoom of red square in (a). (c) Height profile across the 4 nm step highlighted in panel (a). (d) AM-KPFM map of the same area of Figure 5a.

There is an increasing interest in t-2L-LMs and t-ML-LMs, due to their emerging superconducting^49−51^ and correlated insulating behaviors,^52−54^ and in t-ML-TMD where cumulative polarizations have been measured.^31^ We consider a specific region of our t-hBN where an additional layer is present with an uncontrolled orientation relative to the underlaying hBN. This turns the area into a t-ML-hBN sample. Figure 5a is a PFM amplitude map obtained in this zone, related to a topographical step (∼4 nm, see Figure 5c), separating 2 different regions. The top-left part of the image involves a moiré superlattice made of big (Λ_m_ ∼ 300 nm) triangular AB/BA domains. The bottom-right part has two overlapped textures: a first superlattice that follows the previously discussed pattern, plus a second finer superlattice whose tiny details can be visualized through a high-resolution PFM amplitude map, see Figure 5b. Figure 5d is an AM-KPFM map of the same region of Figure 5a. Only the first superlattice can be distinguished, probably due to a weaker IP signal and/or a limited spatial resolution of AM-KPFM.^55^

From Figure 5b, we derive Λ_m_ ∼ 50 nm, smaller than the typical dimension of the first superlattice (Λ_m_ ∼ 300 nm). There is a different geometry of the fine pattern, mainly characterized by hexagonal structures, corresponding to central AA stacking domains. The rounded areas surrounding AA regions may be SP domains, with AB/BA regions limited to very small (but still visible) triangular domains. Considering the ML-hBN structure in this region (schematic in the SI, Section 9), we ascribe this PFM experimental observation to the presence of a double-moiré (in the bottom-right part of Figure 5a), emerging from three t-hBN stacks.

## Conclusions

We used PFM to probe the local electromechanical properties of t-hBN, showing the formation of in-plane polarizations at the edges of the stacking domains (saddle points) of both parallel and antiparallel moiré superlattices. We explained the origin of these saddle point polarizations, proving their universality by evaluating moiré superlattices for a range of twist angles. The relevance of these saddle point polarizations was extended by measuring them also in a double-moiré emerging from the relative twisting of three hBN stacks involving two interfaces.

Our work unveils a richer polarization (in- and out-of-plane) network in t-hBN, whose spatial distribution can be tuned by the twist angle, a behavior not found in conventional bulk ferroelectric materials,^17^ where the polarization domains are determined by the fixed crystal structure. This complex 3D vectorial polarization pattern could trigger interesting topological investigations,^20^ related to negative capacitance,^25^ or high-density information processing,^26^ but also provides new insights for exploring unconventional behaviors in t-LMs. In this context, the experimental observation of a double-moiré is important, due to the properties observed in t-ML-graphene, where superconducting^49−51^ and correlated insulating properties^53,54^ have been found, and in t-ML-TMD, where cumulative polarizations were measured.^31^ Similarly, the emergence of a double-moiré in t-hBN involving both IP and OOP polarizations, could pave the way for moiré ferroelectricity modulations via multistacking.^56^

The ability of PFM to image *both* parallel and antiparallel t-hBN alignments, with high spatial resolution (about 10 nm), not possible with other SPM techniques, confirms it as a very powerful technique to study moiré superlattices in t-LMs.

## Methods

### Sample Fabrication

t-hBN samples are prepared by exfoliating bulk hBN crystals, grown at high pressure and temperature,^57^ onto Si + 90 nm SiO_2_ by micromechanical cleavage (MC). In order to control θ_TW_, either large flakes (>50 μm) selectively torn during transfer^58^ or neighboring hBN flakes cleaved from the same bulk crystal during MC^18^ are identified by studying the orientation of their faceted edges using optical microscopy.^59^ t-hBN samples with controlled interlayer rotation are then fabricated using polycarbonate (PC) stamps.^60^ First, a PC film on polydimethylsiloxane (PDMS) is brought into contact with the substrate with hBN flakes at 40 °C using a micromanipulator, so that the contact front between stamp and substrate covers part of one flake or one of two adjacent flakes exfoliated from the same flake on the tape. Stamps are then retracted, and the material in contact with the PC is picked up from the substrate. After picking up the first flake, a controlled θ_TW_ (±0.01°), as determined by the resolution and wobble of the rotation stage, can be applied by rotating the sample stage, before the flake on PC is aligned to the second one and brought into contact at 40 °C. The stamp is then retracted and the resulting t-hBN is picked up by PC. t-hBN is then transferred onto Si + 285 nm SiO_2_ at 180 °C, before the PC residue is removed by immersion in chloroform and then ethanol for 30 min. While Si + 90 nm SiO_2_ is used to facilitate the identification of hBN flakes,^61^ n-doped Si + 285 nm SiO_2_ is chosen for further characterization, such as gate dependent electrical measurements. Characterizations via Raman spectroscopy is discussed in ref (30), as well as in Section 10 of the SI.

### Scanning Probe Microscopy

AFM measurements are performed at about 25 °C (RH ∼ 40%), in air, using a Multimode 8 (Bruker) AFM microscope. For PFM images we used ASYELEC.01-R2 cantilevers (Asylum Research, *k* ∼ 2.8 N·m^–1^, *f* ∼ 75 kHz). The deflection sensitivity is obtained by performing 10 force–distance curves on mica (without changing the laser spot position onto the cantilever) and calculating the average inverse slope in the contact region. An average value of 68 nm·V^–1^ is found. The nominal tip radius is 25 nm. For an applied force *F = k · d* ∼ 15 nN we calculate, from standard Hertz contact mechanics^62^ and assuming an effective hBN Young modulus ∼35 GPa,^63^ a contact radius *r*_c_ ∼ 2 nm. Considering that the hBN lattice constant is ∼ 0.25 nm,^47^ this contact radius implies a statistical amount of atoms (∼500) involved in the tip–sample interaction, therefore justifying the use of macroscopic parameters, such as polarization and piezoelectric coefficient, also in line with refs (27) and (28).

SCANASYST FLUID cantilevers (Bruker, *k* ∼ 0.7 N·m^–1^, *f* ∼ 150 kHz) are used for phase-imaging, while ASYELEC.01-R2 cantilevers (Asylum Research, *k* ∼ 2.8 N·m^–1^, *f* ∼ 75 kHz) for all the PFM and AM-KPFM images. The phase-imaging typical parameters are free amplitude *A*_0_ ∼ 8 nm, set-point ∼7 nm. The attractive phase values in this work are reported following the Asylum Research convention.^21^ For vertical and lateral PFM, we use a set-point ∼5 nm, with typical contact resonance *f*_CR_ ∼ 330 kHz, and an AC sample bias amplitude *V*_ac_ = 2 V (*V*_dc_ = 0, referring to eq S1). Vertical and lateral PFM measurements for Figure S4 are performed on a Dimension Icon (Bruker) AFM microscope. In AM-KPFM, the images are acquired with *A*_0_ ∼ 20 nm, set-point ∼ 5 nm, lift height ∼ 2 nm, lift driving voltage ∼ 2 V. All AFM images are obtained at a typical scan rate of 0.8 Hz and analyzed in Gwyddion.^48^ AM-KPFM maps are flattened together with a second order polynomial correction to enhance the moiré contrast between AB and BA triangular domains.

## Supporting Information Available

Antiparallel stacking alignments in t-hBN; resonance-enhanced vertical PFM; Figure 2 full set of data; buckling effect; lateral PFM measurements of IP polarizations in t-hBN moiré superlattice; twist angle extraction from PFM images; shape evolution of two antiparallel stacking moiré superlattices; PFM amplitude images for Figure 4; schematic of sample showing a double-moiré in Figure 5a; Raman characterization of the t-hBN sample; and V-PFM images for a 0.8 nm/5.7 nm t-hBN sample (PDF)
