# Supplementary material for: Electromechanical Response of Saddle Points in Twisted hBN Moiré Superlattices
Source: ACS Nano. 2025 Apr 23;19(17):16297–306. doi: 10.1021/acsnano.4c12315 (PMC12060643; doi:10.1021/acsnano.4c12315)
Supplement: Supplementary file 1 — nn4c12315_si_001.pdf [file nn4c12315_si_001.pdf]

Supporting Information

Electromechanical response of saddle points in twisted hBN moiré superlattices

Stefano Chiodini[1], Giacomo Venturi[1], James Kerfoot[2], Jincan Zhang[2], Evgeny M. Alexeev[2], Takashi Taniguchi[3], Kenji Watanabe[4], Andrea C. Ferrari[2] and Antonio Ambrosio[1]

[1]Center for Nano Science and Technology, Fondazione Istituto Italiano di Tecnologia, Via Rubattino 81, 20134, Milan, Italy

[2]Cambridge Graphene Centre, University of Cambridge, 9, JJ Thomson Avenue, CB3 0FA, Cambridge, United Kingdom

[3]Center for Materials Nanoarchitectonics, National Institute for Materials Science, 1-1 Namiki, Tsukuba 305-0044, Japan

[4]Research Center for Functional Materials, National Institute for Materials Science, 1-1 Namiki, Tsukuba 305-0044, Japan

Section 1: Anti-parallel stacking alignments in twisted hexagonal boron nitride (t-hBN)

In the anti-parallel stacking alignment AA', AB' and BA' lattice registries can form (Figure S1) together with saddle points regions (Figure 1a). AA' domains are characterized by the lowest stacking energy, $\Delta\varepsilon$, with AB' and BA' domains described by higher (and different) energy values.[1] Hence, for the anti-parallel stacking, atomic relaxation favors[2-4] AA' regions, with AB' and BA' domains having a minor coverage along the moiré superlattice. AA' stacking has a hexagonal shape,[2, 3] while AB'/BA' domains are more triangular and develop an out-of-plane (OOP) charge density which is three orders of magnitudes weaker than in parallel stacking (AB/BA domains).[2, 3]

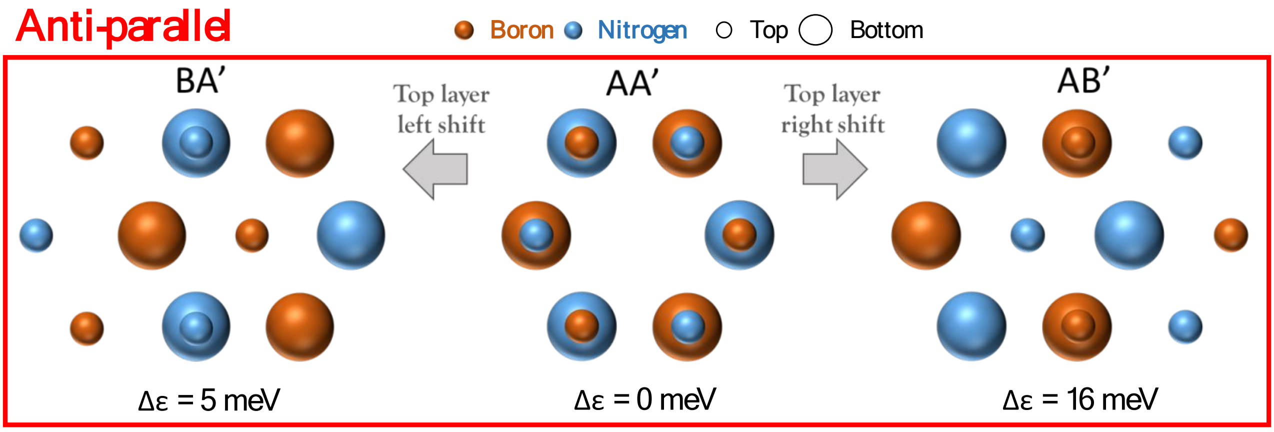

Figure S1. *Stacking domains for anti-parallel alignment in t-hBN. Starting from the natural configuration of hBN, AA' (centre of the figure), two other stacking can exist with different* $\Delta\varepsilon$ *with respect to AA' domains: BA' (left) or AB' (right). B and N atoms of top (smaller circles) and bottom (larger circles) layers are sketched in maroon and blue, respectively.*

Section 2: Resonance-enhanced vertical piezo force microscopy (V-PFM)

Through standard PFM, or the more advanced dual AC resonance tracking[5] and band excitation[6] techniques, a plethora of different samples have been studied, from ferroelectric materials[7] to bio-samples,[8] perovskites,[9, 10] and layered material (LM) moiré superlattices from graphene,[11, 12] to transition metal dichalcogenides (TMDs)[13] and hBN.[12]

We visualize the electromechanical (EM) response of t-hBN samples by applying resonance-enhanced V-PFM to circumvent the issue of materials with a weak (smaller than the cantilever thermal noise) EM response. This technique relies on electrically driving (see below) the cantilever at the contact resonance frequency,[14] in order to boost the signal-to-noise-ratio through resonance amplification. It provides better results for flat surfaces (such as those of LMs) where, due to a small roughness (or root-mean-square (RMS), see Figure S2a) the contact resonance frequency can be considered nearly constant.

When the sample is piezoelectric (PZ), we expect a non-zero PZ tensor relating the applied potential to the sample deformation (referred to as the "inverse piezo effect")[9], which results in an induced polarization. The most relevant component of this tensor for typical PFM studies is the effective piezoelectric coefficient $d_{33,\mathrm{eff}}$, considered as an effective parameter since different EM contributions can determine its magnitude (not only PZ, but also other terms such as electrostatics), together with the cantilever dynamics.[15] In a PFM measurement, the voltage applied at the tip (or sample) is:[16]

$$V_{tip} = V_{dc} + V_{ac}\sin(2\pi f t) \qquad \text{(S1)}$$

causing a sample deformation, traced by the cantilever periodic motion in time (t):[16]

$$z = z_{dc} + A(f, V_{dc}, V_{ac})\sin(2\pi f t + \varphi), \qquad \text{(S2)}$$

where A is the PFM amplitude and φ the phase, measured with demodulating lock-in. In resonance-enhanced PFM, f is equal to the contact resonance frequency (f_{CR}).[14] Thus, eq S2 becomes:

$$z = d_{33,eff}\, V_{dc} + d_{33,eff}\, V_{ac}\, Q\, sin\,(2\pi f_{CR} t + \varphi) \qquad \text{(S3)}$$

where Q is the quality factor of the amplitude resonance curve at f_{CR}.[14]

When the sample is EM active, the PFM amplitude contains information on the magnitude of the effective PZ coefficient,[9] while the PFM phase allows the determination of the polarization direction.[9] $\varphi = 0°$ means that the polarization is parallel to the applied electric field, while for $\varphi = 180°$ the local sample polarization and the electric field are anti-parallel.

Section 3: Figure 2 full set of data (topography, trace and retrace of PFM amplitude and phase)

Figure S2a provides the topography corresponding to Figure 2 of the main text. The *z*-scale bar and the RMS confirm the flatness of the surface. Figure S2b-e compare the PFM amplitude and phase channels acquired during a full pass of the AFM tip along each line of the image (trace in panels (b, c) and retrace in panels (d, e)). The amplitude and phase signals at the borders of the triangle domains overlap, providing a first general indication about the reliability of the PFM measurements. Indeed, despite the quality of Figure S2b-e, there are two unexpected observations regarding the amplitude and phase values. First, there is a phase contrast < 180°, unlike what is expected for opposite in-plane (IP) polarization directions. Second, the amplitude values do not always have a zero-minimum.[11, 15, 17] A similar behavior was reported in Refs.[12, 18]. This inconsistency may rise from a constant background imposed on top of the *real* signal[11]. In the most general case, this background emerges from the interplay between several phenomena, *e.g.* piezoelectricity, electrostatics, electrochemistry, and Joule heating, which cannot be controlled.

Regarding electrostatic contributions coming from the body of the cantilever, the electrostatic blind spot (ESBS) technique[15] provides a convenient way to reduce it. However, this approach is effective only for off-resonance PFM. Due to a weak EM response of the t-hBN sample, our measurements were all performed on-resonance. Additionally, another technique employed to reduce electrostatic artifacts is the interferometric detection system (IDS),[9] a setup not available in all commercial AFM microscopes. In our case, electrostatic contributions are not expected to be dependent on the relative orientation between the cantilever main axis and the side of any triangular moiré domain, because any artifact coming from each saddle point (width ≈ 10 nm, see Figure 2b) would be averaged due to their much smaller extension compared to the cantilever body (hundreds of microns). Therefore, this would correspond to an offset, common to each moiré domain.

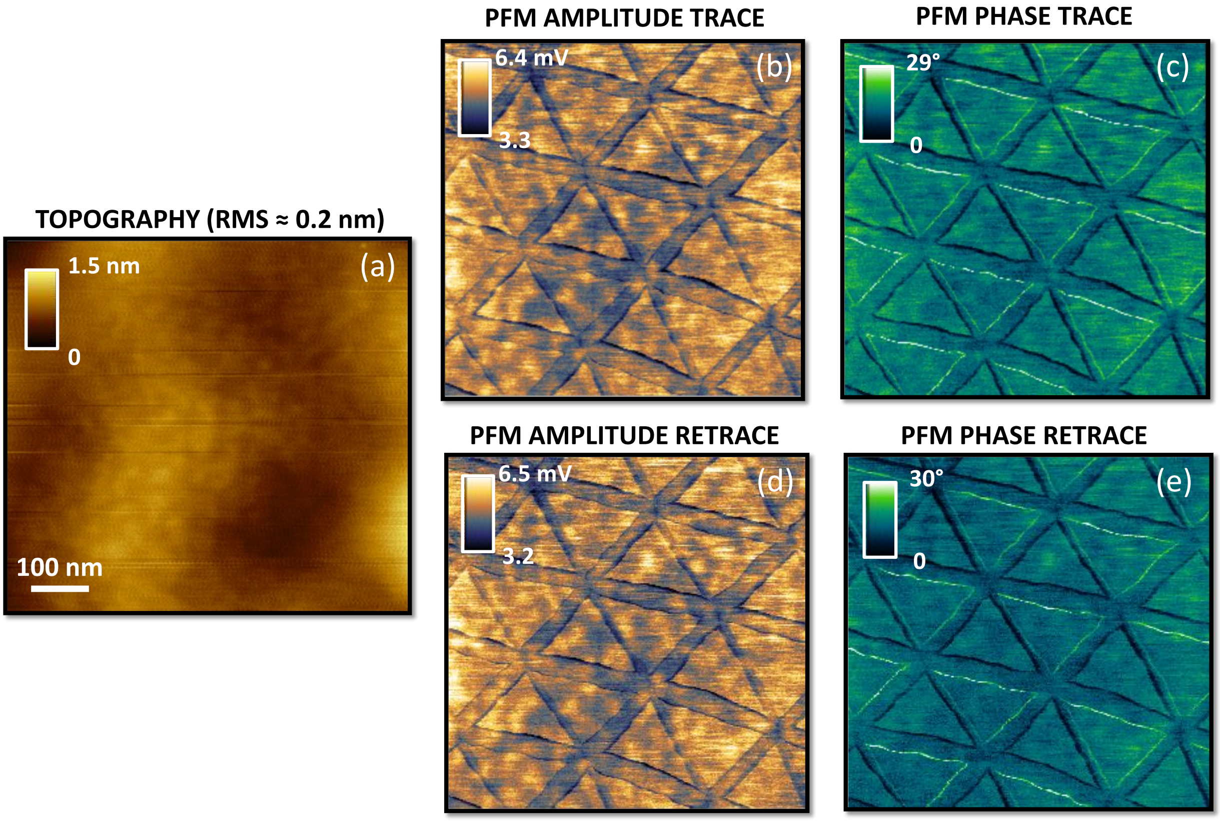

Figure S2. *(a) Topography corresponding to PFM amplitude and phase channels of Figure2. (b, c) Trace PFM amplitude and phase channels. (d, e) Retrace PFM amplitude and phase maps.*

Section 4: Buckling effect

The buckling effect[19, 20] stems from the cantilever buckling oscillations that take place when domains with IP polarization are aligned parallel to the long axis of the cantilever itself. Figure S3 shows the 3 possible orientations between the cantilever long axis and the sample polarizations. Figure S3a reports the most standard case where OOP polarization regions are probed. Accordingly, the cantilever will bend vertically (the tip is displaced up and down, following the sample) providing a vertical signal on the photodiode. If we switch from OOP to IP polarizations, 2 situations can emerge: torsion and buckling. When the sample polarization is perpendicular to the cantilever main axis, a torsional motion of the cantilever occurs due to the IP deformation of the sample that drags the tip. This results in a horizontal photodiode signal (Figure S3b). On the other hand, when the sample polarization is aligned with the cantilever long axis, the tip is dragged by the sample parallel to the axis, causing the buckling oscillation of the cantilever (Figure S3c). As in the first case, a vertical motion of the laser spot onto the photodiode is observed. For this reason, the buckling effect is also known as an in-plane flexural crosstalk.

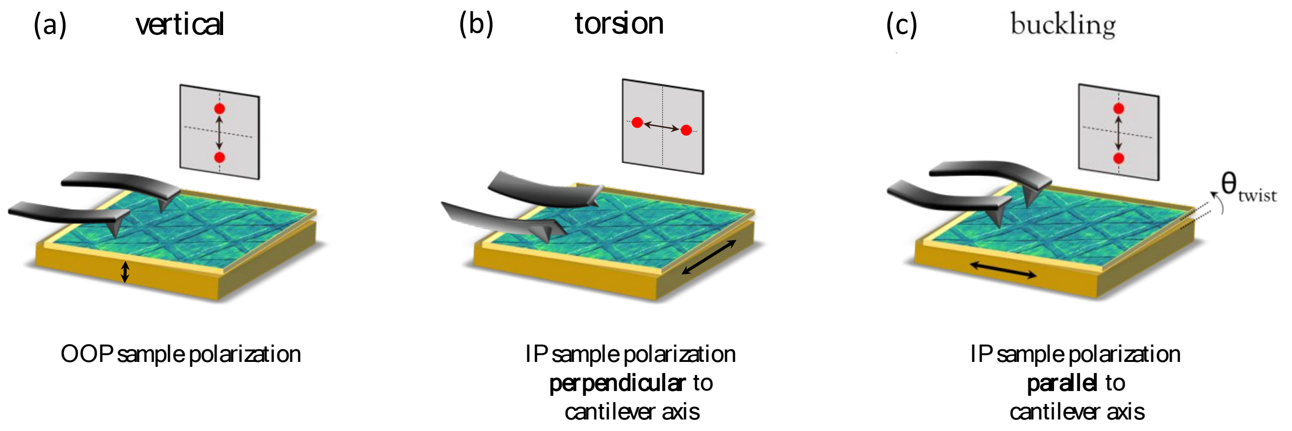

Figure S3. *(a) Schematic of OOP sample polarization causing vertical deflection of the cantilever, i.e. vertical movement of laser spot on the photodiode. (b) Representation of the torsional case, where an IP sample polarization is perpendicular to cantilever long axis. A lateral (horizontal) movement of laser spot is read on the photodiode. (c) Buckling case characterized by an IP sample polarization parallel to the cantilever axis. As in (a), the photodiode reads a vertical signal.*

Section 5: Lateral PFM (L-PFM) measurements of IP polarizations in t-hBN moiré superlattice

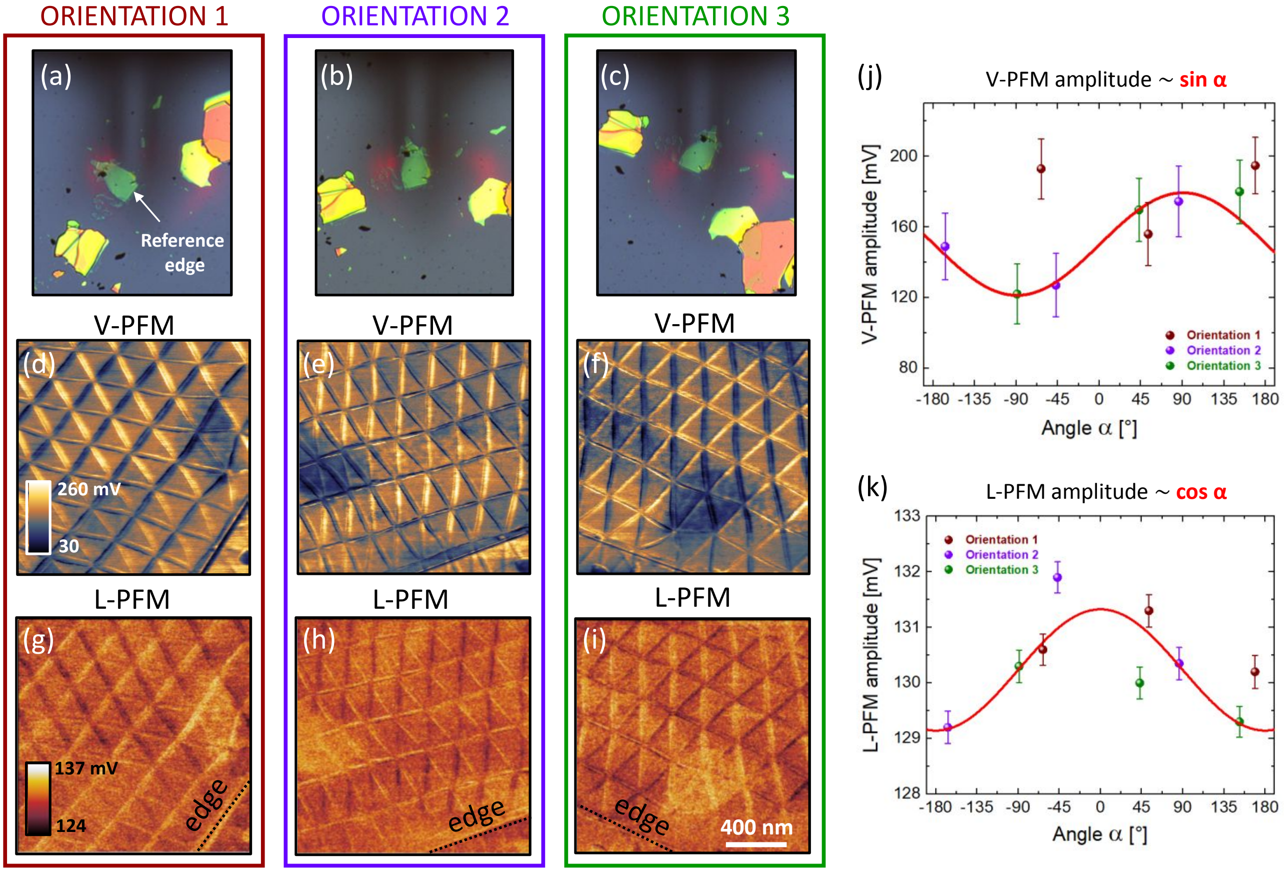

Figure S4. *L-PFM images of a t-hBN moiré pattern. (a, d, g) Optical image, amplitude V-PFM and L-PFM maps, respectively, for a first cantilever-sample orientation as in panel (a) and labelled "Orientation 1". In panel (a) the reference t-hBN edge under investigation is highlighted. (b, e, h) Optical image, amplitude V-PFM and L-PFM maps for a second cantilever-sample orientation as (b) and labelled "Orientation 2". (c, f, i) Optical image, V-PFM and L-PFM maps for a third cantilever-sample orientation as (c) and labelled "Orientation 3". In panels (g, h, i) the position of the edge marked in panel (a) is highlighted with a black dashed line. (j) V-PFM average amplitude along the moiré triangles' 3 sides as a function of the angle α between each side and the axis orthogonal to the cantilever. In maroon, violet and green the data referred to Orientation 1, 2 and 3, respectively. Red line: sinusoidal fitting following the formula:* $A_V = y_{0V} + a_V \cdot \sin\alpha$, *with* y_{0V} *and* a_V *as fitting parameters. (k) L-PFM average amplitude along the 3 moiré triangles' sides as a function of the angle α between the side and the axis orthogonal to the cantilever. In maroon, violet and green the 3*

data referred to Orientation 1, 2 and 3, respectively. Red line: a sinusoidal fitting following the formula: $A_L = y_{0L} + a_L \cdot \cos\alpha$*, with* y_{0L} *and* a_L *as fitting parameters. For each of the 3 orientations, the corresponding polarizations along the edge of a triangular moiré domain are oriented clockwise.*

In Figure S4 we show the results of scanning, by means of V-PFM and L-PFM, a t-hBN region characterized by a parallel moiré superlattice. In order to increase the statistics, we repeat the experiment for 3 different cantilever-sample orientations, *i.e.* "orientation 1, 2 and 3" (see later). In Figure S4d, g we report the V-PFM and L-PFM amplitude maps for the "orientation 1" case, in Figure S4e, h for "orientation 2" and in Figure S4f, i for "orientation 3". For all the 3 orientations, we image the same area characterized by a reference edge (see Figure S4a), which we also highlight in Figure S4g-i with a black dashed line. For each of the 3 cases, we average the V-PFM (A_V) and L-PFM (A_L) amplitude value along the 3 sides of 10 triangular domains, obtaining a total of 9 data points. The same is done for the angle α between the x-axis (Figure 2e) and the polarization associated with the side under consideration (orientation of the polarizations along the perimeter: clockwise).

In Figure S4j we illustrate the plot relating the V-PFM amplitude (extracted from Figure S4d-f) along with the angle α, for each of the 3 orientations. All the 9 values are nicely fit by a sinusoidal function, *i.e.* $A_V = y_{0V} + a_V \cdot \sin\alpha$ (as for Figure 2d data, with more points), where y_{0V} and a_V are the fitting parameters. This confirms the moiré polarizations under consideration to emerge from the buckling effect (SI, Section 4) and have an IP nature. Notably, in Figure S4k, we extend the same analysis to the L-PFM amplitude maps. In this case, all the 9 data are properly fit by the sinusoidal function $A_L = y_{0L} + a_L \cdot \cos\alpha$ with y_{0L} and a_L as fitting parameters. The change from a sine to a cosine stems from the fact that, while the buckling effect is sensitive to the projection of the polarizations along the main cantilever axis (y-axis, Figure 2e), in L-PFM the torsional movement of the cantilever couples with the x-axis projection.

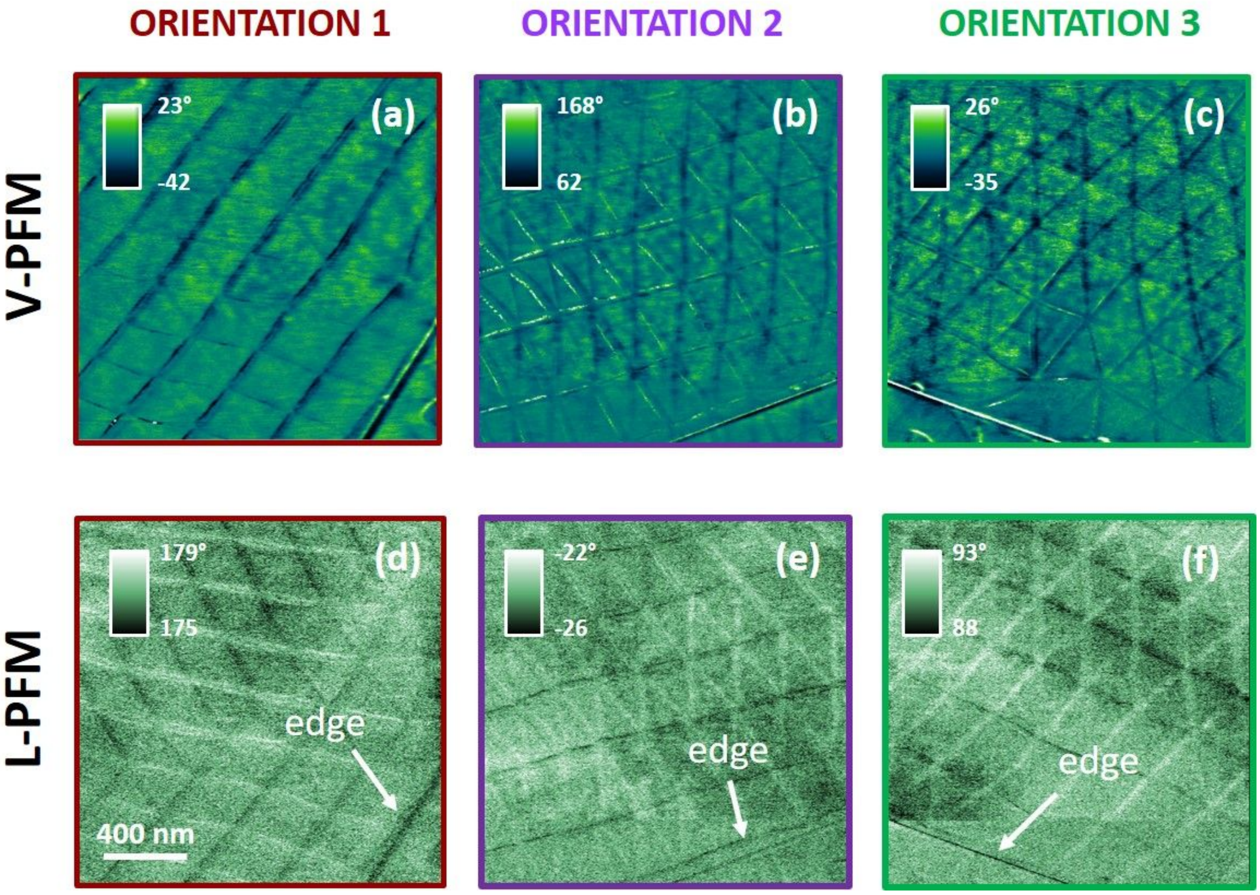

Figure S5. *V-PFM and L-PFM phase images corresponding to the PFM amplitude maps shown in Figure S4d-i.*

In Figure S5, we illustrate the PFM phase images corresponding to Figure S4. Specifically, in Figure S5a-c we show the V-PFM phase images related to Figure S4d-f, while in Figure S5d-f, we report the L-PFM phase images corresponding to Figure S4g-i. The three "orientations" mentioned in Figure S5 (1, 2 and 3) are the same as for Figure S4. Remarkably, in all these images (Figure S5a-f), the IP polarizations are visible. The position of the marked edge can help the comparison with Figure S4.

Section 6: Twist angle extraction from PFM images

As mentioned in the main text, the actual twist angle θ_{TW} of a twisted LM may not be the same θ_{TW} at which two layers are superimposed. First, local imperfections of individual top and bottom LM flakes cause the formation of localized strain difference between the two, which alters the alignment of the atoms, causing a variable-size moiré pattern over the whole interface area between the twisted crystals. On top of this, at $\theta_{TW} \leq 1°$, an additional displacement of the atoms is caused by atomic relaxation, which affects the saddle point regions.[21] Therefore, higher strain is expected at those regions. Simulations involving density functional theory and molecular dynamics are required to compute the new equilibrium position of the displaced atoms in the spatially varying stacking configuration imposed by the layer twisting.[22] However, this does not affect the overall periodicity of the moiré pattern. Notably, after rigid twisting of the layers, the regions characterized by a local AA stacking are not influenced by atomic relaxation.[23] Hence, it is possible to ignore atomic relaxation if the aim is to retrieve the real local θ_{TW}.

The procedure to extract θ_{TW} (and strain tensor) from an experimental image of a spatially varying moiré superlattice was recently described in Ref. [24] We report here the main results that we implement in our derivation of θ_{TW} to support Figure 3 of the main text.

Let us consider two different 1L-hexagonal lattices vertically stacked with a relative in-plane rotation θ as in Figure S6, with unit cell vectors of lengths a_1 and a_2, respectively. The orientations of the two lattices are referred to the x-axis. We assume that the vector connecting neighbouring A sites of the bottom lattice (red arrow) forms an angle ϕ_0 (measured counter-clockwise). By defining the following unit vectors (unit vectors for a hexagonal lattice)

$$\boldsymbol{b_1} = \begin{pmatrix} \cos\phi_0 \\ \sin\phi_0 \end{pmatrix}, \ \boldsymbol{b_2} = \begin{pmatrix} \cos\left(\phi_0 + \frac{\pi}{3}\right) \\ \sin\left(\phi_0 + \frac{\pi}{3}\right) \end{pmatrix},$$

we can write the positions of all the A sites of the bottom layer (blue-shaded circles) as

$$\boldsymbol{r_A^b} = a_1[\boldsymbol{b_1} \quad \boldsymbol{b_2}]\begin{pmatrix} m_1 \\ n_1 \end{pmatrix},$$

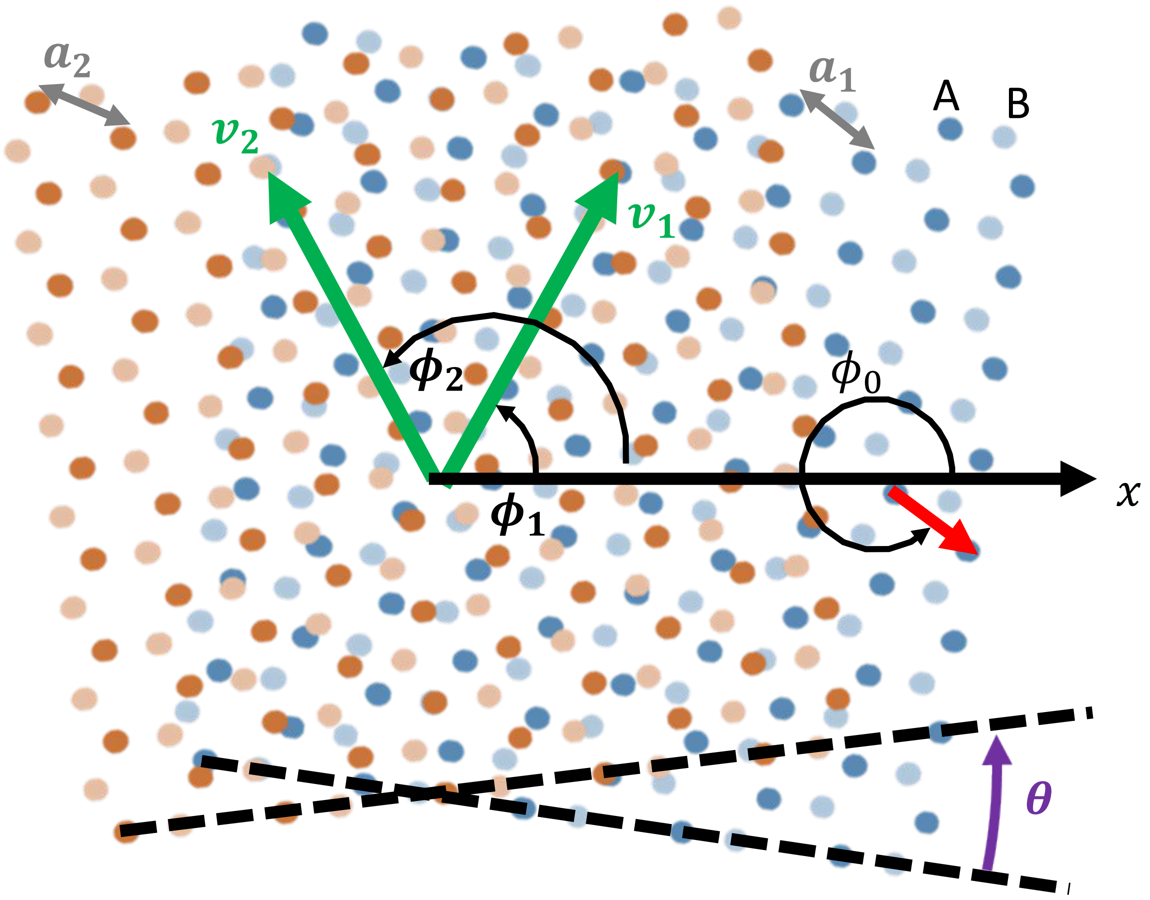

Figure S6. *Schematic representation of a moiré superlattice emerging from two hexagonal lattices (orange and blue shaded dots representing the A and B sites of the crystal) twisted by an in-plane angle θ. The two lattices have different lattice parameters a_1, a_2 (grey). Green arrows: moiré unit cell vectors v_1, v_2. Red arrow: primitive vector of bottom lattice (blue-shaded dots) connecting neighbouring A sites. All the angles are measured counter-clockwise with respect to the x-axis.*

where m_1, n_1 are integers (representing the number of lattice sites away from the origin, which is set on one of the A sites). The same can be done for the position of the A sites in the top layer, by considering the inter-layer rotation (through the rotation matrix $R_\theta = \begin{pmatrix} \cos\theta & -\sin\theta \\ \sin\theta & \cos\theta \end{pmatrix}$) and the presence of a position-dependent displacement ($\hat{\Sigma}\boldsymbol{r}$, where $\hat{\Sigma}$ is the strain matrix):

$$\boldsymbol{r}_{\boldsymbol{A}}^{\boldsymbol{t}} = R_\theta a_2 [\boldsymbol{b}_1 \quad \boldsymbol{b}_2] \binom{m_2}{n_2} + \hat{\Sigma}\boldsymbol{r},$$

for integer values of m_2, n_2. In this case, all the strain (assumed to be uniform) is encoded in the top layer, while the bottom lattice is assumed to be non-deformed. This does not change the results, as the meaningful quantity is the strain difference between top and bottom layers, which alters the atoms alignment, in contrast to a common strain term.[24] Given the above definitions, it is possible to express the lattice sites of the moiré superlattice in terms of $\boldsymbol{r_A^b}$ and $\boldsymbol{r_A^t}$. Due to the hexagonal shape of the two layers, the emerging moiré pattern is also characterized by a hexagonal lattice with unit vectors:

$\boldsymbol{v_1} = \lambda_1(\cos\phi_1, \sin\phi_1)$ and $\boldsymbol{v_2} = \lambda_2(\cos\phi_2, \sin\phi_2)$,

with orientations measured with respect to the x-axis. In absence of strain, $\lambda_1 = \lambda_2$ as there is no asymmetry in the two lattices, however, in general, the two values are different. According to Ref. [24], an expression for the moiré lattice vectors is:

$$[\boldsymbol{v_1} \quad \boldsymbol{v_2}] = a_2\left(R_{-\theta}\left(I - \hat{\Sigma}\right) - (1+\delta)I\right)^{-1}[\boldsymbol{b_1} \quad \boldsymbol{b_2}]$$

where I is the 2x2 identity matrix and $\delta = \frac{a_2}{a_1} - 1$. By rewriting this, we end up with an expression for the strain matrix in terms of θ, ϕ_0, and the experimentally observable parameters $\lambda_1, \lambda_2, \phi_1, \phi_2$:

$$\hat{\Sigma} = I - R_\theta\left(a_2[\boldsymbol{b_1} \quad \boldsymbol{b_2}][\boldsymbol{v_1} \quad \boldsymbol{v_2}]^{-1} + (1+\delta)I\right).$$

By imposing that $\hat{\Sigma}$ is θ-independent (*i.e.*, $\hat{\Sigma}_{12} = \hat{\Sigma}_{21}$), as we are neglecting any atomic relaxation term,[24] we find the parametric relationship (ϕ_0 is the parameter) between θ_{TW} and the observable parameters $\lambda_1, \lambda_2, \phi_1, \phi_2$:

$$e^{i\theta(\phi_0)} = \frac{1}{R(\phi_0)}(x(\phi_0) + iy(\phi_0)) \tag{S6}$$

with:

$$x(\phi_0) = x_0 + r_- \cos(\alpha_- - \phi_0)$$

$$y(\phi_0) = r_- \sin(\alpha_- - \phi_0)$$

$$R(\phi_0) = \sqrt{x(\phi_0)^2 + y(\phi_0)^2}$$

where

$$\Delta\phi = \phi_2 - \phi_1,$$

$$x_0 = \frac{2(1+\delta)}{a_2}\lambda_1\lambda_2 \sin\Delta\phi\,,$$

$$r_\pm = \sqrt{\lambda_1^2 + \lambda_2^2 - 2\lambda_1\lambda_2\cos\left(\Delta\phi \mp \frac{\pi}{3}\right)},$$

$$e^{i\alpha_\pm} = \frac{i}{r_\pm}\left(\lambda_1 e^{i\left(\phi_1 \pm \frac{\pi}{3}\right)} - \lambda_2 e^{i\phi_2}\right).$$

As a result, $\hat{\Sigma}$ is a symmetric matrix that can be written as:

$$\hat{\Sigma} = \epsilon_c I + \epsilon_s \begin{pmatrix} \cos\gamma & \sin\gamma \\ \sin\gamma & -\cos\gamma \end{pmatrix}$$

where ϵ_c and ϵ_s represent the isotropic compression and shear strain terms, respectively, and γ defines the strain direction:

$$\epsilon_s = \frac{a_2 r_+}{2\lambda_1\lambda_2 \sin\Delta\phi} \tag{S7}$$

$$\epsilon_c(\phi_0) = 1 - \frac{1+\delta}{x_0}R(\phi_0) \tag{S8}$$

$$e^{i\gamma(\phi_0)} = e^{i(\theta(\phi_0)+\alpha_+ + \phi_0 + \pi)}. \tag{S9}$$

Eq. S6-S9 allow us to extract information about the (local) θ_{TW} and strain values from the knowledge of geometrical quantities of a moiré pattern, *i.e.*, size and shape of the superlattice. However, as emerges from the parametric dependence of θ, ϵ_c and γ, this assumes ϕ_0 to be known, otherwise multiple solutions can exist. In most cases the exact orientation of the bottom layer is not known. Nevertheless, a practical way to choose a proper value for ϕ_0 is the one that minimizes ϵ_c^2 (as suggested in Ref. [24]).

As most of LM crystals have an in-plane hexagonal lattice, the described framework is rather general, however, here we focus on the extraction of θ_{TW} and strain from an experimental image of a t-hBN moiré superlattice: hence we set $a_1 = a_2 = a = 0.25$ nm and $\delta = 0$.

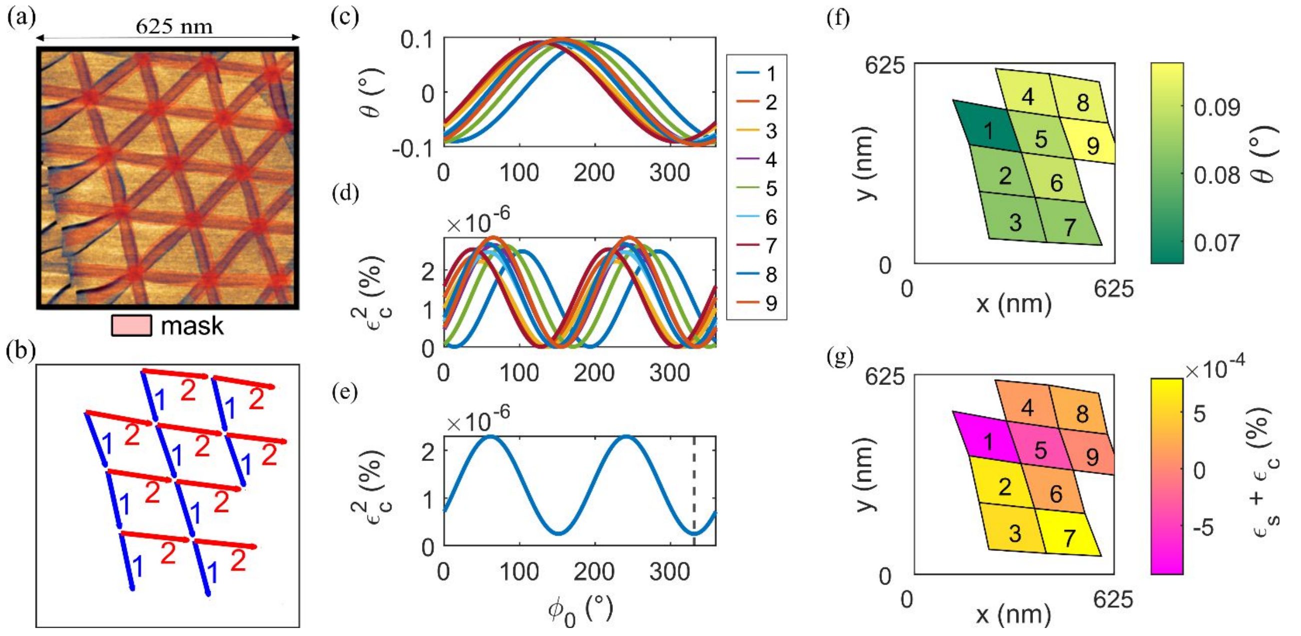

Figure S7. *Procedure for the extraction of the local twist angle and strain from an image of a moiré pattern. (a) PFM map of t-hBN moiré pattern (Figure 3c of the main text) with a mask to filter for the perimeter of each triangular moiré domain. (b) Local lattice vectors (1 and 2) corresponding to each moiré domain fully enclosed in the image (9 domains are defined this way). Values of the twist angle (c) and square of the compressive strain (d) as a function of the bottom lattice orientation* ϕ_0 *for each moiré domain defined by the lattice vectors in (b). (d) Average value of* ϵ_c^2 *performed on all the 9 domains (labelled in (f), (g)). Dashed line indicates the minimum (in the π-2π range) that fixes the value for* ϕ_0*. (f, g) Map of the twist angle and total strain, respectively, corresponding to the image in (a), showing the position-dependent values.*

In real-case scenarios, measured moiré patterns are irregular across a sufficiently large (tens of unit cells) detection area, *i.e.*, $\boldsymbol{v_1}$,$\boldsymbol{v_2}$ are functions of the position, hence also the strain matrix and twist angle. Therefore, all the above formulas are valid only locally (assuming an infinite pattern with the same local size and orientation).

To retrieve θ,[24] our starting point is a typical PFM map, as in Figure S7a. The triangular moiré domains change across the image, meaning that eq. S6-S9 hold for each triangle individually, which in turns has different values of θ_{TW} and strain. We first apply a mask (using the edge detection

function in Gwyddion)[25] to isolate the perimeter of each triangular domain of the measured moiré superlattice. From this mask, we can extract the intersection points representing the AA stacking regions (this is done with the help of a Radon transform in Matlab, which allows for the identification of the direction of each line of the mask). The distance from neighbouring AA sites defines the moiré lattice vectors for each domain, as shown in Figure S7b, where all the extracted couples $(\boldsymbol{v_1}, \boldsymbol{v_2})$ are shown (we consider only AA sites for which the neighbouring sites fall within the image boundaries, 9 in total). These provide the experimental (local) values for $\lambda_1, \lambda_2, \phi_1, \phi_2$. For each of the 9 moiré unit cells defined by the vectors in (b), we compute θ and ϵ_c^2 as a function of the unknown parameter ϕ_0, see Figure S7c, d. These functions are slightly different because of the variation of the $\boldsymbol{v_1}, \boldsymbol{v_2}$ vectors in the image. We fix ϕ_0 by minimizing the average ϵ_c^2 for the entire image, shown by the dashed line in Figure S7e (in doing this, ϵ_s is ignored since is ϕ_0-independent). The resulting values for the extracted θ_{TW} and total strain as a function of position in the imaged area are shown in Figure S7f, g, respectively. Each moiré cell (uniform since atomic relaxation is ignored) is characterized by the corresponding θ and $\epsilon_s + \epsilon_c$ at the target ϕ_0.

The main result is that the values for θ are very close to θ_{TW} derived for an unstrained lattice: the average value for Figure S7f is 0.086°, which only differs by 7% from the 0.093° for the unstrained case. We repeated the same extraction procedure also for the other images shown in Figure 3, finding differences no larger than 7%, thus validating the values reported in the main text.

Regarding the total strain, instead, we have very small values compared to what is found, *e.g.*, in Ref. [24] for a moiré pattern that presents a morphological deformation caused by the fabrication process (a bubble). This is due to the flatness of the scanned areas in Figure 3, for which the strain is ~ 0.0001%. This approach does not consider atomic relaxation (the displacement is assumed to be continuously varying through the term $\hat{\Sigma}\boldsymbol{r}$), thereby the real value for the total amount of strain may be underestimated.[26]

Section 7: Shape evolution of two anti-parallel stacking moiré superlattices

FigureS8 shows two moiré superlattices of an anti-parallel t-hBN sample (top flake ≈ 4.5 nm, bottom layer thickness ~ 40 nm, θ_{TW}~ 0.2°). These two regions are characterized by a different local θ_{TW} and, therefore, Λ_m, due to fabrication imperfections. Both PFM amplitude and phase maps display moiré patterns typical of anti-parallel stacking, confirming that AA' hexagonal domains occupy most of the superlattice, as they are energetically favoured (Figure S1). Figure S8a, c show PFM signals from a region where Λ_m~200 nm. Figure S8b, d plot the PFM signals of a different region of the same t-hBN, where the moiré superlattice is characterized by a smaller Λ_m~100 nm. As observed in Figure 3 of the main text, if Λ_m gets smaller (from Figure S8a to b), the relative area covered by the less energetically favoured domains (here, AB'/BA') should increase (opposite trend for AA' hexagonal regions). This is seen in Figure S8.

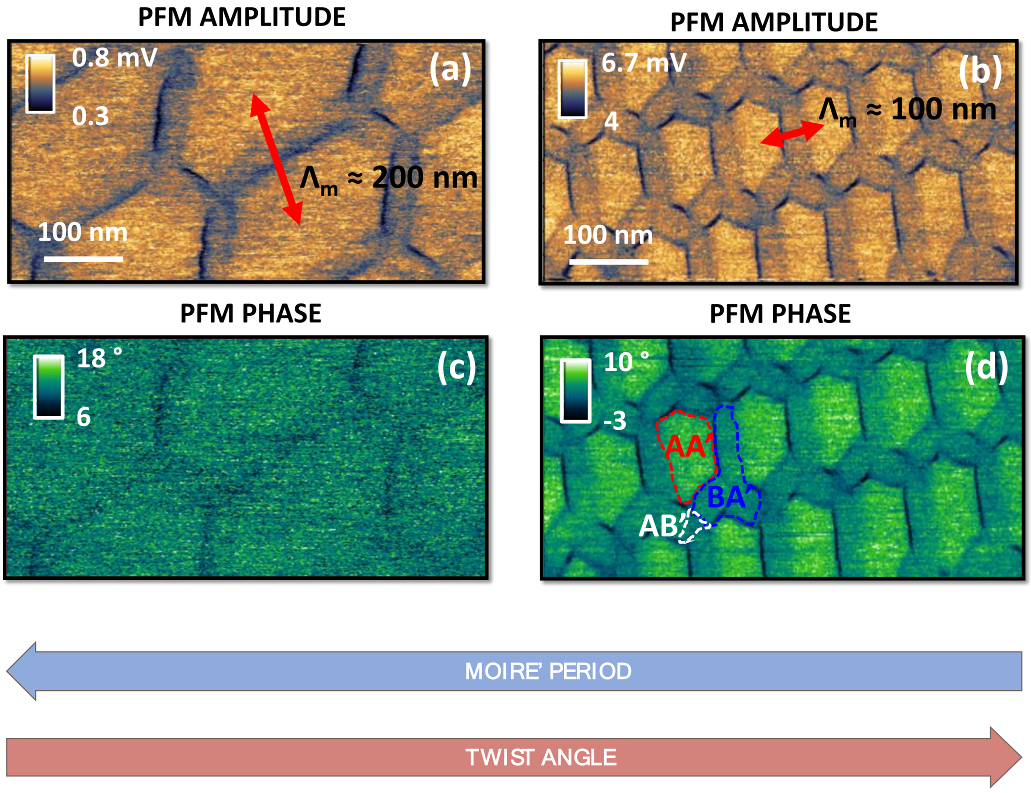

Figure S~~7~~8. *(a, c) PFM amplitude and phase images of anti-parallel moiré superlattice with Λ_m~200 nm (red arrow). (b, d) PFM amplitude and phase maps of smaller moiré superlattice with Λ_m~100 nm (red arrow). In (d) the positions of AA' (red), BA' (blue) and AB' (white) stacking are indicated.*

Section 8: PFM amplitude images for Figure 4 of main text

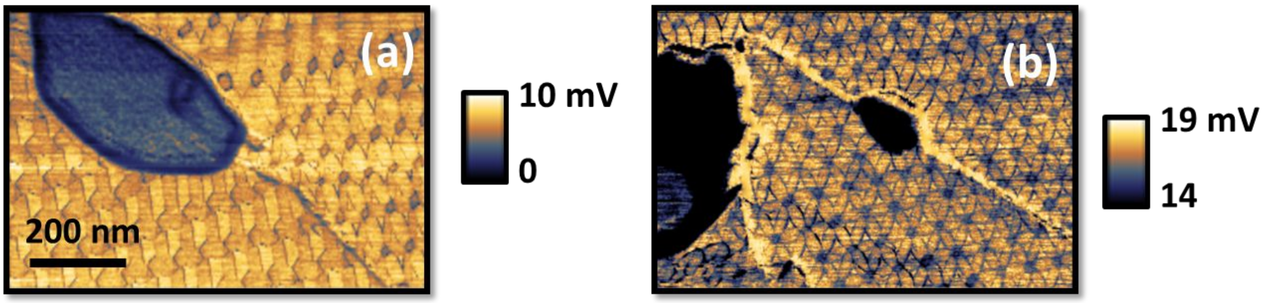

Figure S~~8~~9. *(a) PFM amplitude map related to PFM phase map of Figure 4b. (b) PFM amplitude image related to PFM phase map of Figure 4g.*

Section 9: Schematic of sample showing a double-moiré in Figure 5a.

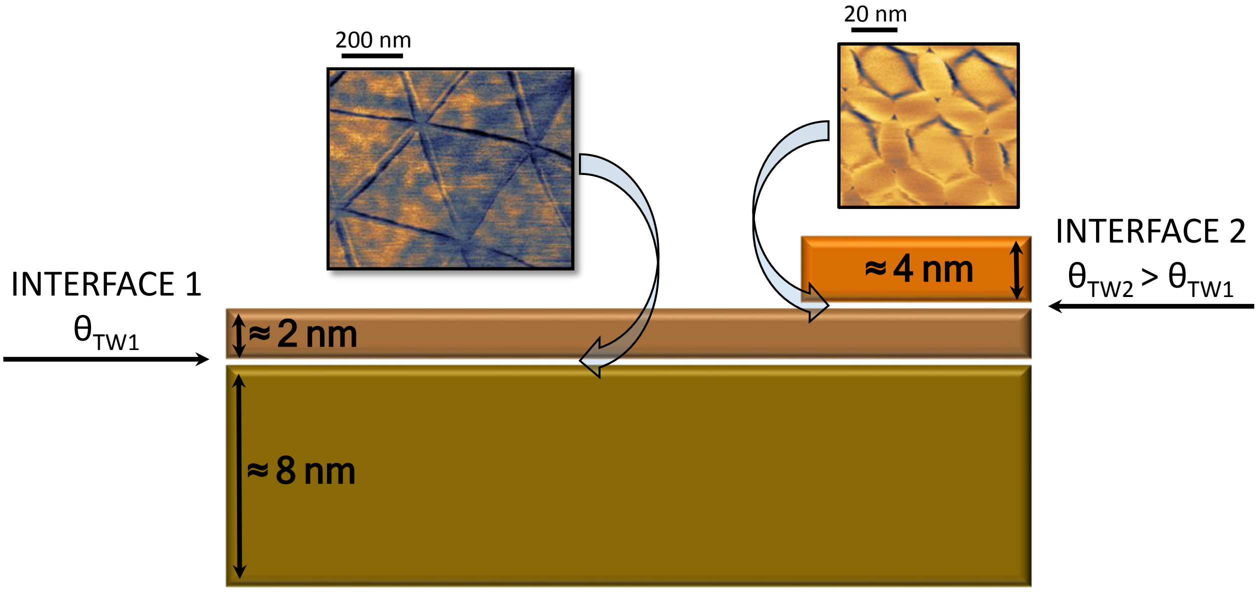

Figure S910. *Schematic t-hBN sample providing a double-moiré in Figure 5. 3 hBN flakes are present with 2 interfaces (1 and 2). Correspondingly, two moiré superlattices are present. At "interface 2" they overlap, providing a double-moiré pattern.*

Section 10: Raman characterisation of the t-hBN sample

To monitor the quality and track any induced strain and disorder within the hBN heterostructures throughout fabrication, Raman spectroscopy is performed using a Horiba LabRAM Evolution at 514 nm, with an 1800 l/mm grating and volume Bragg filters with a ~5 cm^{-1} cut-off frequency and a 100x objective (NA: 0.9). Errors associated with the peak position and full width at half maximum (FWHM) are systematically explored by taking into account fitting error, spectrometer registry and statistical and transient variations[13]. In Ref. [27] we characterised the Raman spectra of the t-hBN referred to in Figure 2, 3a, b and 5. Raman spectroscopy is used to confirm the thickness of top and bottom flakes *via* the position of the shear mode (C). Pos(C) can be used to determine the layer number (N), for N > 2, [14,15,16] with N = 5 extracted for the top hBN flake and N>10 for the bottom flake. The flakes thicknesses are ~ 2 and ~ 8 nm by atomic force microscopy (AFM), in agreement with Raman spectroscopy.

Raman spectroscopy is also performed on hBN on native Si + SiO_2, as for Figure 3c-e and 4. The thickness of the bottom flake is~43.7nm, while the top flake varies from 1L-hBN to up to 6.5 nm, as confirmed by AFM. Figure S11a plots the Raman spectra of one of the bottom hBN flake. Owing to the differing intensity of reflections from the native Si versus Si+90nm SiO_2 of Ref. [27], we observe a significant increase in the ultra-low frequency background, attributed to reflected light from the 514 nm laser. The background is subtracted by fitting the spectra to an exponential function $A_1 e^{\frac{x-x_0}{c_1}} + A_2 e^{\frac{x-x_0}{c_2}}$, as shown by a red line in Figure S11a. The fitted background is subtracted to give the spectra in grey in Figure S11a. Figure S~~10~~11b, c plot the Raman spectra of the bottom hBN flake, t-hBN and the starting bulk hBN (B-hBN). For the bottom 43.7 nm hBN, t-hBN on Si and the starting B-hBN: Pos(C) = 52.4 ± 0.14 cm^{-1}, with FWHM(C) = 1 cm^{-1} ± 0.2 cm^{-1}. Pos(C) can be used to determine N, for N > 2, [14,15,16]

$$\text{Pos(C)} = \frac{1}{\sqrt{2}\pi c}\sqrt{\frac{\alpha_{\perp}}{\mu}}\sqrt{1+\cos\left(\frac{\pi}{N}\right)} \qquad \text{(S6)}$$

With c the speed of light in cm s^{-1}, $\mu = 6.9 \times 10^{-27}$ kg·Å^{-2} the mass of one layer per unit area and $\alpha_{\perp}$ the interlayer coupling. [13, 14, 15] From eq. S6, we estimate N > 10 for the bottom hBN and t-hBN. Figure S11c gives Pos (E_{2g}) = 1365.2 ± 0.2 cm^{-1} with FWHM (E_{2g}) = 7.4 ± 0.2 cm^{-1} for 43.7 nm, t-hBN, and B-hBN, which implies that the strain across the bottom flake and the t-hBN is <0.007 % based on the E_{2g} shift rate[17]. Notably, this strain evaluation should not be considered perfectly accurate due to a diffraction limited Raman spot which necessarily takes into account many t-hBN moiré domains.

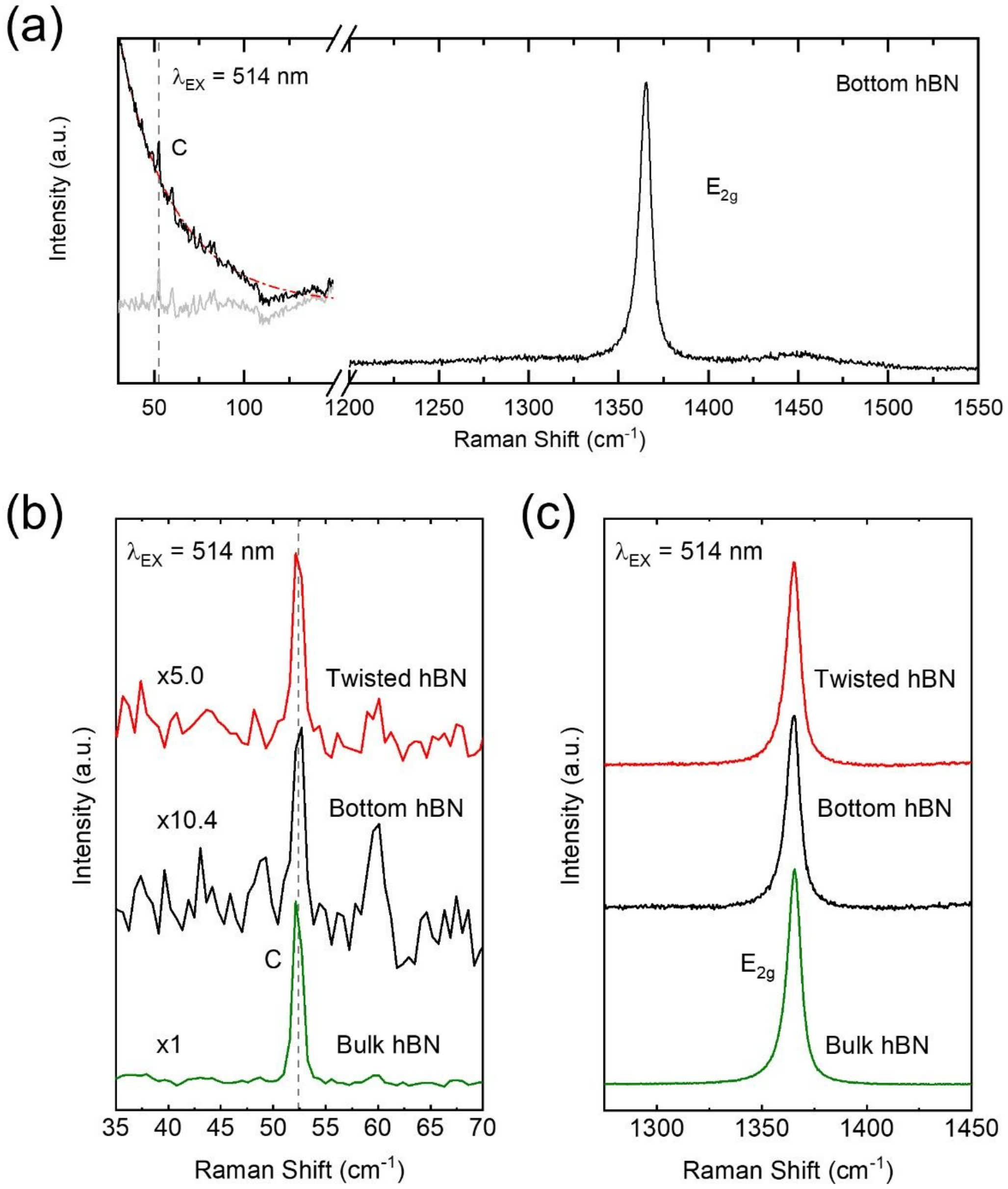

Figure S11. *(a) Raman spectra of bottom 43.7 nm hBN on native Si is shown in black alongside the fit to the ultra-low frequency background, red. (b) Corrected low frequency spectrum. Low and (c)*

high-frequency Raman spectra of t-hBN (red), 43.7nm hBN (black) on Si and B-hBN (green). The spectra in (b, c) are normalised relative to I(C) and I(E_{2g}).

Section 11: V-PFM images for a 0.8 nm/5.7 nm t-hBN sample

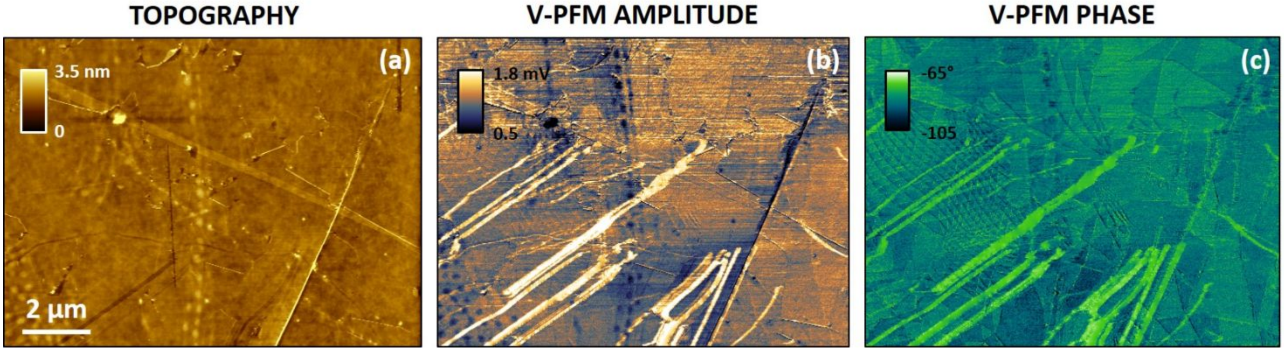

Figure S12. *(a) Topography of a 0.8 nm/5.7 nm t-hBN sample region. (b, c) Corresponding V-PFM amplitude and phase channels.*

In Figure S12 we report the V-PFM images on a t-hBN sample characterized by a thinner top flake with respect to the sample investigated in Figure 2. Specifically, we refer to a 0.8 nm-thick top hBN with 5.7 nm of bottom flake ($\theta_{TW} \approx 0.2°$). Figure S12a shows the flat topography of a large area. Figure S12b, c, instead, correspond to the V-PFM amplitude and phase maps of the same region. Notably, despite the low image-quality, the contrast between adjacent AB/BA triangular domains is visible, especially in the phase map.

References

1. Constantinescu, G.; Kuc, A.; Heine, T., Stacking in Bulk and Bilayer Hexagonal Boron Nitride. *Phys. Rev. Lett.* **2013,** *111,* 036104.
2. Walet, N. R.; Guinea, F., Flat Bands, Strains, and Charge Distribution in Twisted Bilayer Hbn. *Phys. Rev. B* **2021,** *103,* 125427.
3. Woods, C. R.; Ares, P.; Nevison-Andrews, H.; Holwill, M. J.; Fabregas, R.; Guinea, F.; Geim, A. K.; Novoselov, K. S.; Walet, N. R.; Fumagalli, L., Charge-Polarized Interfacial Superlattices in Marginally Twisted Hexagonal Boron Nitride. *Nat. Commun.* **2021,** *12,* 347.
4. Ribeiro, R. M.; Peres, N. M. R., Stability of Boron Nitride Bilayers: Ground-State Energies, Interlayer Distances, and Tight-Binding Description. *Phys. Rev. B* **2011,** *83,* 235312.

5. Rodriguez, B. J.; Callahan, C.; Kalinin, S. V.; Proksch, R., Dual-Frequency Resonance-Tracking Atomic Force Microscopy. *Nanotechnology* **2007,** *18,* 475504.
6. Jesse, S.; Kalinin, S. V.; Proksch, R.; Baddorf, A. P.; Rodriguez, B. J., The Band Excitation Method in Scanning Probe Microscopy for Rapid Mapping of Energy Dissipation on the Nanoscale. *Nanotechnology* **2007,** *18,* 435503.
7. Kolosov, O.; Gruverman, A.; Hatano, J.; Takahashi, K.; Tokumoto, H., Nanoscale Visualization and Control of Ferroelectric Domains by Atomic Force Microscopy. *Phys. Rev. Lett.* **1995,** *74,* 4309-4312.
8. Li, J.; Li, J.-F.; Yu, Q.; Chen, Q. N.; Xie, S., Strain-Based Scanning Probe Microscopies for Functional Materials, Biological Structures, and Electrochemical Systems. *J. Materiomics* **2015,** *1,* 3-21.
9. Collins, L.; Liu, Y.; Ovchinnikova, O. S.; Proksch, R., Quantitative Electromechanical Atomic Force Microscopy. *ACS Nano* **2019,** *13,* 8055-8066.
10. Kim, H.-S.; Kim, S. K.; Kim, B. J.; Shin, K.-S.; Gupta, M. K.; Jung, H. S.; Kim, S.-W.; Park, N.-G., Ferroelectric Polarization in Ch3nh3pbi3 Perovskite. *J. Phys. Chem. Lett.* **2015,** *6,* 1729-1735.
11. Li, Y.; Wang, X.; Tang, D.; Wang, X.; Watanabe, K.; Taniguchi, T.; Gamelin, D. R.; Cobden, D. H.; Yankowitz, M.; Xu, X.; Li, J., Unraveling Strain Gradient Induced Electromechanical Coupling in Twisted Double Bilayer Graphene Moiré Superlattices. *Adv. Mater.* **2021,** *33,* 2105879.
12. McGilly, L. J.; Kerelsky, A.; Finney, N. R.; Shapovalov, K.; Shih, E.-M.; Ghiotto, A.; Zeng, Y.; Moore, S. L.; Wu, W.; Bai, Y.; Watanabe, K.; Taniguchi, T.; Stengel, M.; Zhou, L.; Hone, J.; Zhu, X.; Basov, D. N.; Dean, C.; Dreyer, C. E.; Pasupathy, A. N., Visualization of Moiré Superlattices. *Nat. Nanotechnol.* **2020,** *15,* 580-584.
13. Wang, X.; Yasuda, K.; Zhang, Y.; Liu, S.; Watanabe, K.; Taniguchi, T.; Hone, J.; Fu, L.; Jarillo-Herrero, P., Interfacial Ferroelectricity in Rhombohedral-Stacked Bilayer Transition Metal Dichalcogenides. *Nat. Nanotechnol.* **2022,** *17,* 367-371.
14. Hurley, D. C., Contact Resonance Force Microscopy Techniques for Nanomechanical Measurements. In *Applied Scanning Probe Methods Xi: Scanning Probe Microscopy Techniques*, Bhushan, B.; Fuchs, H., Eds. Springer Berlin Heidelberg: Berlin, Heidelberg, 2009; pp 97-138.
15. Killgore, J. P.; Robins, L.; Collins, L., Electrostatically-Blind Quantitative Piezoresponse Force Microscopy Free of Distributed-Force Artifacts. *Nanoscale Adv.* **2022,** *4,* 2036-2045.
16. Jesse, S.; Baddorf, A. P.; Kalinin, S. V., Dynamic Behaviour in Piezoresponse Force Microscopy. *Nanotechnology* **2006,** *17,* 1615-28.
17. Kim, S.; Seol, D.; Lu, X.; Alexe, M.; Kim, Y., Electrostatic-Free Piezoresponse Force Microscopy. *Sci. Rep.* **2017,** *7,* 41657.
18. Yasuda, K.; Wang, X.; Watanabe, K.; Taniguchi, T.; Jarillo-Herrero, P., Stacking-Engineered Ferroelectricity in Bilayer Boron Nitride. *Science* **2021,** *372,* 1458-1462.
19. Alikin, D. O.; Gimadeeva, L. V.; Ankudinov, A. V.; Hu, Q.; Shur, V. Y.; Kholkin, A. L., In-Plane Polarization Contribution to the Vertical Piezoresponse Force Microscopy Signal Mediated by the Cantilever "Buckling". *Appl. Surf. Sci.* **2021,** *543,* 148808.
20. Nath, R.; Hong, S.; Klug, J. A.; Imre, A.; Bedzyk, M. J.; Katiyar, R. S.; Auciello, O., Effects of Cantilever Buckling on Vector Piezoresponse Force Microscopy Imaging of Ferroelectric Domains in Bifeo3 Nanostructures. *Appl. Phys. Lett.* **2010,** *96,* 163101.
21. Quan, J.; Linhart, L.; Lin, M.-L.; Lee, D.; Zhu, J.; Wang, C.-Y.; Hsu, W.-T.; Choi, J.; Embley, J.; Young, C.; Taniguchi, T.; Watanabe, K.; Shih, C.-K.; Lai, K.; MacDonald, A. H.; Tan, P.-H.; Libisch, F.; Li, X., Phonon Renormalization in Reconstructed Mos2 Moiré Superlattices. *Nat. Mater.* **2021,** *20,* 1100-1105.
22. Vu, T.-H.-Y.; Bennett, D.; Nadeera Pallewella, G.; Hemayet Uddin, M.; Xing, K.; Zhao, W.; Huat Lee, S.; Mao, Z.; Muir, J. B.; Jia, L.; Davis, J. A.; Watanabe, K.; Taniguchi, T.; Adam, S.; Sharma, P.; Fuhrer, M. S.; Edmonds, M. T. J. a. e.-p. Imaging Topological Polar Structures in Marginally Twisted 2d Semiconductors **2024**, arXiv:2405.15126. https://ui.adsabs.harvard.edu/abs/2024arXiv240515126V (accessed May 01, 2024).
23. Cazeaux, P.; Luskin, M.; Massatt, D., Energy Minimization of Two Dimensional Incommensurate Heterostructures. *Arch. Ration. Mech. Anal.* **2020,** *235,* 1289-1325.
24. Halbertal, D.; Shabani, S.; Passupathy, A. N.; Basov, D. N., Extracting the Strain Matrix and Twist Angle from the Moiré Superlattice in Van Der Waals Heterostructures. *ACS Nano* **2022,** *16,* 1471-1476.
25. www.gwyddion.net (accessed accessed September 2022).

26. Halbertal, D.; Finney, N. R.; Sunku, S. S.; Kerelsky, A.; Rubio-Verdú, C.; Shabani, S.; Xian, L.; Carr, S.; Chen, S.; Zhang, C.; Wang, L.; Gonzalez-Acevedo, D.; McLeod, A. S.; Rhodes, D.; Watanabe, K.; Taniguchi, T.; Kaxiras, E.; Dean, C. R.; Hone, J. C.; Pasupathy, A. N.*, et al.*, Moiré Metrology of Energy Landscapes in Van Der Waals Heterostructures. *Nat. Commun.* **2021,** *12,* 242.
27. Chiodini, S.; Kerfoot, J.; Venturi, G.; Mignuzzi, S.; Alexeev, E. M.; Teixeira Rosa, B.; Tongay, S.; Taniguchi, T.; Watanabe, K.; Ferrari, A. C.; Ambrosio, A., Moiré Modulation of Van Der Waals Potential in Twisted Hexagonal Boron Nitride. *ACS Nano* **2022,** *16,* 7589-7604.
